# Identification of tumor antigens and immune subtypes in breast cancer for mRNA vaccine development

**DOI:** 10.3389/fonc.2022.973712

**Published:** 2022-09-26

**Authors:** Ruo Qi Li, Wei Wang, Lei Yan, Li Ying Song, Xin Guan, Wei Zhang, Jing Lian

**Affiliations:** ^1^ Department of Pathology, Cancer Hospital Affiliated to Shanxi Province Cancer Hospital/Shanxi Hospital Affiliated to Cancer Hospital, Chinese Academy of Medical Sciences/Cancer Hospital Affiliated to Shanxi Medical University, Taiyuan, China; ^2^ General Surgery Department, Third Hospital of Shanxi Medical University, Shanxi Bethune Hospital, Shanxi Academy of Medical Sciences, Taiyuan, China; ^3^ Department of Urologic Surgery, Shanxi Medical University Second Affiliated Hospital, Taiyuan, China; ^4^ Department of Orthopaedic Surgery, Shanxi Medical University Second Affiliated Hospital, Taiyuan, China; ^5^ Thyroid Surgery Department, First Hospital of Shanxi Medical University, Taiyuan, China; ^6^ Cardiovascular Department, Third Hospital of Shanxi Medical University, Shanxi Bethune Hospital, Shanxi Academy of Medical Sciences, Tongji Shanxi Hospital, Taiyuan, China; ^7^ Department of Urologic Surgery, First Hospital of Shanxi Medical University, Taiyuan, China

**Keywords:** breast cancer, tumor antigen, immune subtypes, mRNA vaccine, tumor immune infiltration

## Abstract

**Background:**

Poor prognosis, resistance to chemotherapy, insensitivity to radiotherapy, and a high prevalence of adverse drug reactions remain urgent issues for breast cancer (BC) patients. Increased knowledge of tumor immunobiology and vaccine development suggests the possibility of cancer vaccination. Here, we investigated potential BC-associated antigens for the development of an anti-BC mRNA vaccine and populations suitable for mRNA vaccination.

**Methods:**

Gene expression and clinical data were obtained from The Cancer Genome Atlas (TCGA) and Molecular Taxonomy of Breast Cancer International Consortium (METABRIC). The single-cell sequencing data were obtained from the Single Cell Portal platform. cBioPortal was used to visualize and compare genetic alterations. Correlations between immune cell infiltration and antigen expression were visualized with the Tumor Immune Estimation Resource (TIMER). Immune subtypes were identified by consensus clustering and analysis of immune infiltration. Biomarkers for the assessment of mRNA vaccination suitability were investigated.

**Results:**

Three tumor-associated antigens, CD74, IRF1, and PSME2, that showed overexpression, amplification, and mutation and were linked with prognosis and immune cell infiltration, were identified. Single-cell sequencing analysis showed the expression of the three tumor-associated antigens in different cells of BC. Three immune subtypes were identified among BC patients, with Cluster B patients having a tumor microenvironment conducive to immunotherapy. These subtypes also showed different expression patterns of immune checkpoints, immune cell death-promoting genes, and response to immune checkpoint inhibitor (ICI) therapy. Thus, we identified five biomarkers that could be applied for assessing vaccination suitability and predicted drugs that would be appropriate for patients unsuited for vaccination.

**Conclusions:**

Our findings suggest new directions for the development of mRNA vaccines against breast cancer.

## Introduction

Worldwide, the “pink killer” breast cancer (BC) represents about 30% of cancers in women, with a high mortality (1). The cost of surgery, hospitalization, loss of productivity, and emotional trauma place a heavy burden on the patient’s follow-up and treatment, as well as adding insecurity and economic burden to society (2). There are multiple subtypes of BC, classified according to their gene expression profiles. Four subtypes, namely, luminal A and B, HER2-overexpressing, and basal-like, are the best categorized (3). Despite progress in early detection and treatment, leading to a 38% in mortality rate, many patients have a poor prognosis (4). For example, the five-year survival rate for basal-like BC is 84%, with overall survival rates averaging only one year for patients with stage IV disease, lower than other BC subtypes (5). In addition, some patients do not respond to chemotherapy or radiotherapy or may experience severe side effects, including damage to the nervous system (6). It is thus necessary to investigate and develop different treatments.

Cancer immunotherapy includes the use of monoclonal antibodies (mAbs), cell therapies, lytic virus treatment, and vaccination, amongst others, and offers great promise for the treatment of a variety of tumor types (7). It is well-known that the immune system plays a key role in BC (8). Antagonists targeting the immune checkpoints CTLA-4 and programmed cell death protein 1 (PD-1) and its ligand 1 (PD-L1) have achieved satisfactory results in a large number of clinical trials and appear to induce long-lasting responses that benefit overall survival (OS) in BC (9). For example, treatment with the anti-PD-1 mAb pembrolizumab resulted in an objective response rate of 18.5% in 27 triple-negative BC (TNBC) patients previously treated with chemotherapy (10). Besides, cancer vaccines are becoming increasingly attractive to oncologists as an emerging hotspot for BC immunotherapy.

The rationale behind tumor vaccines is the induction or amplification of tumor-associated T cells that target and destroy the tumor (11). The mRNA vaccines have several benefits over other vaccine types, including ease of preparation, non-integration into the host’s genome, and their possible destruction by cellular RNases, which increase their overall safety (12). Recent research has suggested the possibility of using mRNA vaccines to combat several malignancies. The safety of the new mRNA vaccine has been demonstrated in patients with metastatic gastrointestinal (GI) cancer, where the presence of CD8+ and CD4+ neoantigen-specific T cells stimulated by the vaccine were detected (13). A recent preclinical study demonstrated the efficacy of an mRNA vaccine targeting Trp2 that resulted in an antigen-specific T cell response in melanoma (14). A phase I/II clinical trial of two mRNA vaccines (CV-9103 and CV-9104) for prostate cancer targeting four prostate-specific antigens (STEAP, PSCA, PSMA, and PSA) produced good results (15). Combination immunotherapy using an mRNA vaccine against MUC1 and an anti-CTLA-4 mAb was found to be highly effective in comparison with either the vaccine or the mAb alone in TNBC patients (16). Although there are relatively few studies on the use of mRNA vaccines against BC, the concept of immunization against BC-specific tumor-associated antigens (TAAs) of BC is feasible and worthy of investigation. However, none of the studies have explored the possibility of immune mRNA vaccines in BC.

Here, a systematic analysis of BC sequencing data was performed. Three TAAs that correlated with good patient prognosis and tumor infiltration by antigen-presenting cells were identified. This allowed the stratification of BC patients into three immune categories based on differences in molecular, cellular, and clinical characteristics. It is hoped that these results will offer a valuable reference for the further development and administration of cancer vaccines, as well as for determining the optimal combination of immunotherapies for specific patients.

## Methods

### Data sources

**Supplementary Figure S1** shows the workflow of the study. Data on gene expression (fragments per kilobase million, FPKM), patient prognosis, and clinicopathology were obtained from The Cancer Genome Atlas (TCGA) (https://portal.gdc.cancer.gov/). The final analysis included 1104 samples and the detailed clinical information on these 1104 patients was extracted (**Supplementary Table S1**). Additional data on 1904 cases were obtained from the METABRIC (Molecular Taxonomy of Breast Cancer International Consortium) dataset in the cBioPortal database (http://www.cbioportal.org/,version v4.1.5). The detailed clinical information on these 1904 patients together with OS data are shown in **Supplementary Table S2**. The summaries of the sample compositions of the different clinical groups are provided in **Supplementary Table S3**. The FPKM values from the TCGA BC (TCGA-BRCA) cohort were converted to transcripts per million (TPM) before normalization. The mutect2-processed BC mutation dataset from TCGA was also used and data on 1726 immune-associated genes, including cytokines, interferons, interleukins, and TNFs together with their respective receptors were downloaded from the IMMPORT database (https://www.immport.org/shared/genelists). **Supplementary Table S4** lists the information on the immune-related genes.

#### Single cell data collection and quality control

The single-cell sequencing data from three primary BCs were obtained from the Single Cell Portal platform (http://singlecell.broadinstitute.org) (accession number EGAS00001005115). This dataset contained a total of 77,881 BC cells. A Seurat object was created using the “Seurat” package V4.0 (https://satijalab.org/seurat) (17). Cells were further filtered according to the following threshold parameters: the total number of expressed genes, 200–9000; and proportion of mitochondrial genes expressed<20%. The “NormalizeData” function in Seurat was used to normalize the expression matrix of single cells. The “FindVariableFeatures” function in Seurat was used to find the top 2000 highly variable genes. The expression levels of highly variable genes were scaled and centered using the “ScaleData” function in order to exclude the influence of mitochondrial genes. A total of 38,941 annotated BC cells were obtained. These cells were renamed by annotated information from the Single Cell Portal. Data were visualized in two dimensions using the “uniform manifold approximation and projection for dimension reduction” method (18). “DimPlot” function is used to visualize the expression of specific genes in different types of cells.

### Tumor antigen identification by cBioPortal analysis

The cBioPortal for Cancer Genomics (http://www.cbioportal.org) is an online tool for the integration of raw data from large studies on a variety of cancers (19). cBioPortal was used for the exploration, visualization, and analysis of genetic alterations in BC antigens together with multidimensional cancer genomic data. Both mutated genes and amplified genes in BC were obtained from cBioPortal, and the genes are displayed in **Supplementary Table S4**.

### TIMER analysis

The Tumor Immunization Estimation Resource (TIMER, https://cistrome.shinyapps.io/timer/) analyzes immune infiltration data in various tumor types (20). TIMER was used here to determine the relationships between infiltration and tumor-associated antigen expression. Correlations were determined by Spearman’s analysis with Purity Adjustment and P-values < 0.05.

### Identification of immunophenotyping in BC patients

Information on the expression patterns of 1726 immune-associated genes was obtained from the TCGA-BRCA and METABRIC cohorts. According to the gene expression of the OS-related immune gene profiles, the “ConsensusClusterPlus” R package was used for the separation of immune subtypes to find populations suited for vaccination (21). The clustering algorithm used the k-means of Euclidean distance with 50 sub-samplings, each resampling using 80% of the complete sample population, and nine maximum clusters. The optimal K was calculated by the elbow method and ensured that the patient numbers in the individual clusters exceeded 100. The R package “Rtsne” was applied for t-distributed stochastic neighbor embedding (t-SNE) to reduce the data dimensionality and to determine sample distributions after clustering (22). Survival was compared between the clusters using the “survival” R package and the “pheatmap” package (https://CRAN.R-project.org/package=pheatmap) was used for the creation of heatmaps for clinically relevant antigens to illustrate the differences in expression between the populations.

### Gene set variation analysis

Gene set variation analysis (GSVA) was used to investigate the relationships between immune subtype and biological process, using the “GSVA” package in R (23). Functional annotation was performed with “clusterProfiler” and the gene set file (c2.cp.kegg.v7.2.symbols.gmt) was obtained from the MSigDB database (https://www.gsea-msigdb.org) (24, 25).

### Analysis of the tumor infiltration microenvironment

Single-sample gene-set enrichment analysis (ssGSEA) was used to determine the relative abundance of different immune cell types infiltrating the tumor microenvironment (TME) (26). The ImmuneScore, StromalScore, ESTIMATEScore, and TumorPurity were determined by the “ESTIMATE” package. In addition, CIBERSORT was used to calculate the fractions of 22 immune cell types in individual samples (27). Information on immune checkpoint (ICP) and immune cell death (ICD)-modulating genes were acquired from previous publications (12).

### Predicted responses to immunotherapy in the three clusters

Immunotherapy responses were predicted using the Tumor Immune Dysfunction and Exclusion (TIDE) online tool (http://tide.dfci.harvard.edu/) (28). A higher TIDE prediction score represents a greater potential for immune evasion and suggests that the patient is less likely to benefit from immune checkpoint inhibitor (ICI) therapy (28). We used TIDE to evaluate the potential clinical efficacy of immunotherapy in the different clusters.

### Mutation identification

Visualization of gene mutation patterns in the clusters was performed with the “maftool” R package which also calculated the tumor mutation burden and mutation numbers in individual samples (29). Additionally, a set of tumor driver genes for BC were extracted from the NCG database (http://ncg.kcl.ac.uk/index.php). The Human Genome Assembly GRCh38 was utilized as the reference genome (https://www.ncbi.nlm.nih.gov/grc/human/data?asm=GRCh38). Mutations with q-values < 0.05 were considered significant, and mutated driver genes were mapped to chromosomes with the “RCircos” R package (30).

### Weighted gene co-expression network analysis

The Weighted Gene Co-expression Network Analysis (WGCNA) was carried out using the “WGCNA” package in R and normalized immune-related gene expression (31). There were no outlying samples discovered after clustering the samples. Thus, the subsequent analysis included all the samples. Pearson correlation coefficients between genes were calculated and applied for the creation of a similarity matrix. The representation matrix was converted to an adjacent and then to a topological matrix. At least 30 genes were included in each module. Eight modules were identified after placement of the soft threshold at three. Links between the modules and the cluster categories were determined and, finally, the “turquoise” module was considered to have a significant association with the vaccination-suitable population. GO and KEGG functional enrichment of the genes in the “turquoise” module were also assessed with the “Clusterprolifer” package.

### Biomarkers with the Potential to Assess Prognosis after mRNA Vaccination

ROC curves, plotted by the “timeROC” package (https://CRAN.R-project.org/package=timeROC), were used to assess the associations with all genes and prognosis. Genes with area under the curve (AUC) values larger than 0.75 at one and three years, and those with AUCs greater than 0.70 at five years, were considered to be potential biomarkers.

### Anti-cancer drug sensitivity analyses

The sensitivities of 21 documented anti-BC chemotherapeutic agents in the different treatment clusters were assessed with the R package “pRRophetic” and the Genomics of Drug Sensitivity in Cancer (GDSC) database (32, 33).

## Results

### Exploring potential tumor antigens of BC

**Figure 1:**
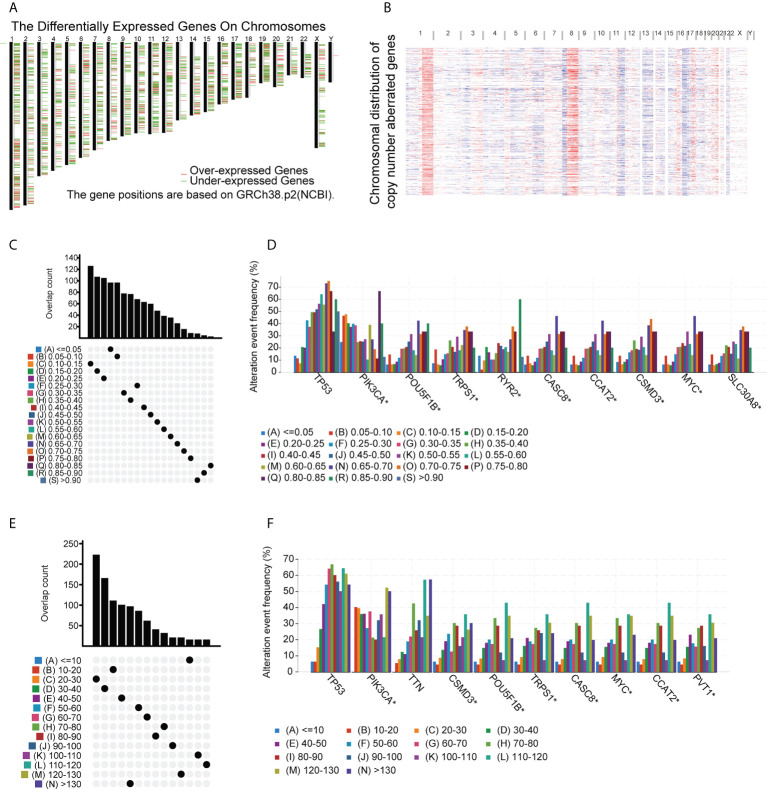
Potential BC tumor-associated antigens. **(A)** Chromosomal locations of differentially expressed genes. **(B)** Chromosomal locations of genes with aberrant copy numbers. **(C-F)** Potential tumor-associated antigens. Overlap between mutated genes in the genome fraction altered group **(C)** and the mutation count group **(E)**. Top genes in the genome fraction altered group **(D)** and mutation count group **(F)**. * P<0.05.

TAAs incorporated into mRNA vaccines should have higher expression in tumors compared with normal tissue (34). In addition, tumors carrying large numbers of mutated TAAs tend to be more susceptible to ICP blockade (35). Therefore, analysis of the tumor mutation profile of individual patients is necessary to generate personalized BC vaccines with high potency. In the initial evaluation of potential TAAs, 18 309 abnormally expressed genes were identified, of which 8824 were upregulated and 9485 were downregulated (**Figure 1A**). An investigation of the copy numbers of the abnormally expressed genes that were likely to encode TAAs (**Figure 1B**). The 16 491 mutated genes and the 21 644 amplified genes are listed in **Supplementary Table S4**. Next, frequently mutated genes that may encode tumor-specific antigens were selected by analyzing the altered genome fraction and mutation counts in each sample. Most BC patients showed relatively high fraction-of-genome alterations and mutation counts (**Figures 1C, E**) indicating that BC has high immunogenicity. It was also found that tumor protein p53 (TP53) and Phosphatidylinositol-4,5-Bisphosphate 3-Kinase Catalytic Subunit Alpha (PIK3CA) had the highest number of mutations determined by both altered genome fractions and mutation counts (**Figures 1D, F**). In addition to TP53 and PIK3CA, other genes including POU Class 5 Homeobox 1B, Transcriptional Repressor GATA Binding 1, Ryanodine Receptor 2, Cancer Susceptibility 8, Colon Cancer Associated Transcript 2, CUB and Sushi Multiple Domains 3, MYC Proto-Oncogene, and Solute Carrier Family 30 Member 8 also showed high degrees of change in the altered genome fraction category (**Figure 1D**) while the 10 top-scoring genes in the mutation-count category were Titin, CUB and Sushi Multiple Domains 3, POU Class 5 Homeobox 1B, Tribbles Pseudokinase 1, Cancer Susceptibility 8, MYC Proto-Oncogene, Colon Cancer-Associated Transcript 2, and Pvt1 Oncogene (**Figure 1F**). Taken together, 4986 upregulated and frequently mutated tumor-associated genes were identified.

### Identification of TAAs linked to prognosis and antigen-presenting cells

**Figure 2:**
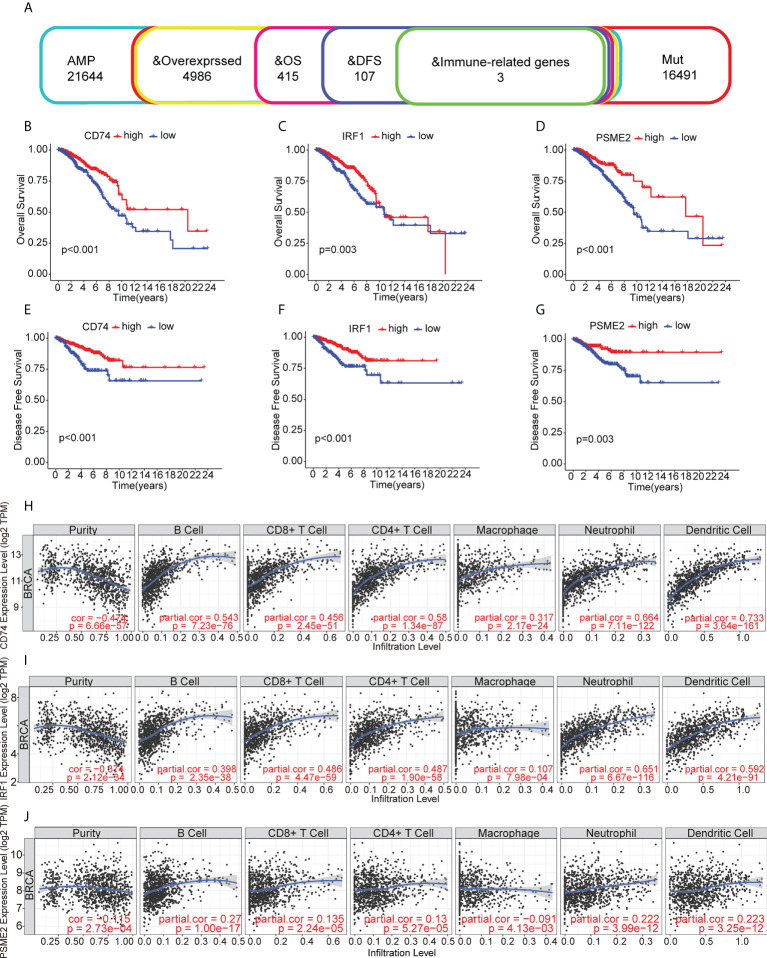
Relationships of tumor-associated antigens with prognosis and immune cells. **(A)** Selection of tumor-associated antigens, based on amplification, mutation, and overexpression (from a total of 4986 candidates), and immune-related genes significantly associated with OS and DFS (total of three candidates). **(B-D)** Kaplan-Meier OS curves for groups with different levels of CD74 **(B)**, IRF1 **(C)**, and PSME2 **(D)**. **(E)** Relationships between CD74 levels and the immune cell proportions and numbers of B cells, CD8+ T cells, CD4+ T cells, macrophages, neutrophils, and dendritic cells. **(E-G)** Kaplan-Meier DFS curves for groups with different levels of CD74 **(E)**, IRF1 **(F)**, and PSME2 **(G)**. **(H)** Relationship between CD74 levels and immune cell proportions and numbers of B cells, CD8+ T cells, CD4+ T cells, macrophages, neutrophils, and dendritic cells. **(I)** Relationship between IRF1 levels and immune cell proportions and numbers of B cells, CD8+ T cells, CD4+ T cells, macrophages, neutrophils, and dendritic cells in BC. **(J)** Relationships between PSME2 levels and the immune cell proportions and numbers of B cells, CD8+ T cells, CD4+ T cells, macrophages, neutrophils, and dendritic cells.

**Figure 3:**
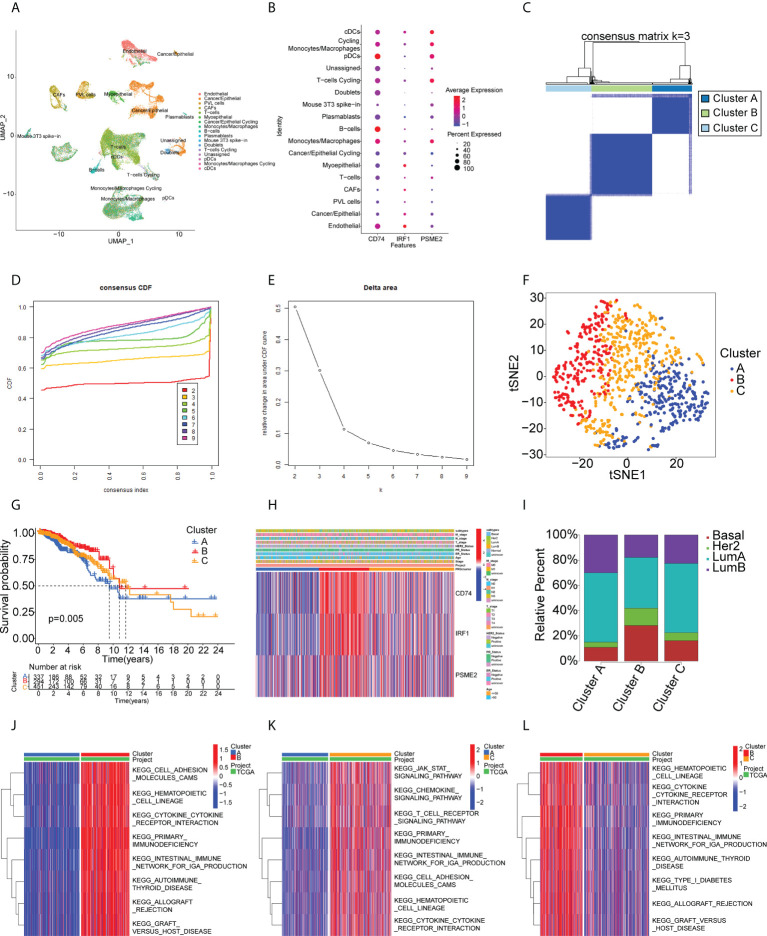
Single-cell transcriptomic profiles from 17 cell subtypes from 3 BC patients and immune clusters of BC patients in the TCGA cohort. **(A)** 2d visualization of 17 cell subtypes in 3 BC patients on the UMAP plot. **(B)** the expression of CD74, IRF1, PSME2 in 17 cell types was visualized by bubble plot. **(C-E)** Three clusters of patients in the TCGA cohort divided according to levels of prognostic immune-related genes. **(F)** t-distributed stochastic neighbor embedding (t-SNE) analysis of distributions in the clusters. **(G)** Survival analysis in the clusters. **(H)** Heatmap of tumor antigen expression and clinical parameters. **(I)** Distribution of immune clusters in patients with different subtypes. **(J-L)** GSVA enrichment analysis showing pathway activation in the clusters. Red color in the heatmap presents pathway activation and blue color represents pathway inhibition.

The identified genes were then evaluated for their relationships with prognosis. This showed that 415 genes were closely associated with BC OS, of which 107 were linked with disease-free survival (DFS). After analysis of the genes’ overlap with 1726 immune-related genes, three specific TAAs, namely, CD74 Molecule (CD74), Interferon Regulatory Factor 1 (IRF1), and Proteasome Activator Subunit 2 were identified (PSME2) (**Figure 2A**). Patients with higher levels of tumor-associated CD74 had significantly better outcomes in comparison with those with lower levels and high levels of IRF1 and PSME2 were also linked with better prognosis (**Figures 2B–G**). Importantly, the expression levels of CD74 (**Figure 2H**), IRF1 (**Figure 2I**), and PSME2 (**Figure 2J**) were positively associated with the levels of most immune cells, such as B cells, CD8+ T cells, CD4+ T cells, neutrophils, and dendritic cells (DCs). Thus, three specific TAAs (CD74, IRF1, and PSME2) were found to be potential vaccine candidates. As these TAAs appear able to stimulate immune activity, they could be processed and presented by antigen-presenting cells to provoke an anti-tumor response. We next explored the expression of CD74, IRF1 and PSME2 in 17 cell types based on single-cell sequencing analysis and visualized the results (**Figure 3A**). The results showed that CD74 expression was relatively high in B cells, monocytes/macrophages and pre-dendritic cells (pDCs), IRF1 expression was relatively high in endothelial cells, myoepithelial cells, and Cancer-associated fibroblasts (CAFs) and PSME2 expression was relatively high in monocytes/macrophages, cycling monocytes/macrophages, cycling T cells and conventional dendritic cells (cDCs) (**Figure 3B**).

### Identification of potential BC immune subtypes

Immunotyping is useful as it reflects the immune status of the tumor and the TME and can thus assist the identification of suitable candidates for vaccination. A total of 1726 immune gene profiles were extracted and subjected to univariate Cox regression analysis, with P<0.05 being chosen as the threshold for screening. Ultimately 281 genes associated with OS were screened for subsequent analysis (**Supplementary Table S5**). The ability of these 281 genes to predict recurrence-free survival (RFS), disease free interval (DFI), and progression free interval (PFI) is also shown in **Supplementary Table S5**. The results showed that most of the screened genes also had the ability to predict RFS, DFI and PFI. Additional enrichment analyses were performed to examine their physiological roles. A consensus cluster was then constructed using the prognosis-related profiles of immune genes. The classifier model was most stable when k was set to 3 (**Figures 3C-D**, **Supplementary Figure S2**). Ultimately, three immune subtypes (Clusters A, B, and C) were obtained with the least within-group minimum variance and between-group maximum variance values (**Figure 3E**). The patient classifications and clinical characteristics of the clusters are also summarized in **Supplementary Tables S6** and **S7**. After t-SNE analysis to reduce the dimensionality of the data, it was found that the three clusters differed significantly (**Figure 3F**) with Cluster B showing the best prognosis (**Figure 3G**). This suggested that the BC TME influenced prognosis and verified both the reproducibility and stability of the results. Notably, CD74, IRF1, and PSME2 were strongly expressed in Cluster B (**Figure 3H**). These findings indicate that Cluster B patients may recruit more immune cells to fight cancer than the rest of the population. This also suggests that Cluster B patients would be likely to benefit from an mRNA vaccine directed against these TAAs. Further analysis of the distribution of patients with different BC molecular subtypes indicated irregular clustering (**Figure 3I**). Both the basal and HER2 subtypes were more common in Cluster B, suggesting patients with these subtypes were more likely to benefit from immunotherapy, consistent with clinical findings (36, 37). Cluster B also showed enrichment in numerous immune-associated pathways, as shown by the GSVA results (**Figures 3J–3L**). Thus, immunotyping is effective for prognostic prediction in BC and it is likely that mRNA vaccines directed against the three identified TAAs may be more effective in patients with basal-like and HER2 BC.

### TME characteristics in relation to immune cluster

**Figure 4:**
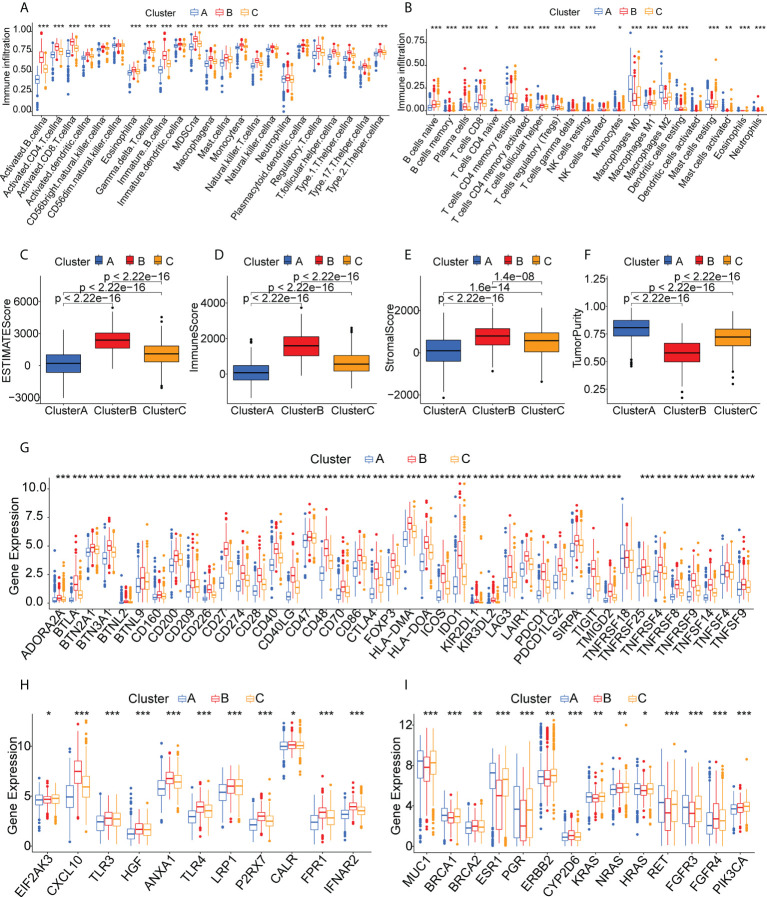
Immune cell infiltration in the different clusters. **(A)** Boxplot showing immune cell infiltration, shown by ssGSEA enrichment. **(B)** Boxplot showing immune cell infiltration, shown by CIBERSORT. **(C-F)** Immune-stromal scores and tumor purity of patients in the clusters, determined by ESTIMATE. **(G)** Expression of immune checkpoints in the clusters. **(H)** Expression of immunogenic cell death modulators in the clusters. **(I)** Comparison of breast cancer biomarkers between the immune clusters. "*" represents that p-value <0.05; "**" represents that p-value <0.01, and "***" represents that p-value <0.001.

The different proportions of immune cells associated with TMEs fell into 28 signatures, shown by ssGSEA analysis (**Figure 4A**). Cluster B had a greater abundance of innate immune cells, including B cells, CD4 +T cells, CD8+ T cells, eosinophils, macrophages, MDSCs, mast cells, and T helper cells (**Figure 4A**). CIBERSORT analysis indicated greater numbers of cytotoxic immune cells (native B cells and CD8 T cells) but fewer immunosuppressive regulatory immune cells (macrophage M0 and macrophage M2 cells) in Cluster B (**Figure 4B**). The immune and stromal scores obtained by ESTIMATE (**Figures 4C–F**) indicated that these were higher in Cluster B, while the tumor purity was lower in this cluster, suggesting higher numbers of tumor-infiltrating immune cells. These findings suggest that Cluster B may contain “hot” immune subtypes, with Cluster C probably is in an intermediate state, and Cluster A may be immunologically “cold”. An earlier study by Thorsson et al. proposed six immune categories (C1-C6) based on an analysis of over 1000 samples from 33 cancers (38). The immune subtypes identified here are also comparable to previously described pan-cancer immune subtypes (**Supplementary Figure S3**). The individual immune categories varied considerably in their proportions in the three subtypes. For example, C1 (wound healing) was predominantly concentrated in Cluster A and C, whereas C2 (IFN-r) was predominantly concentrated in Cluster B. C4 (immune-quiet) was not present in the Cluster B population, suggesting that the C4 immune category may be less sensitive to RNA vaccines, thus complementing previous findings. Both ICPs and ICD modulators play vital roles in controlling the immune response to the tumor as well as minimizing damage. The differential expression of these genes in the three subtypes (**Figures 4G, H**) showed that they were expressed strongly in Cluster B, a further indication that these patients may respond well to mRNA vaccines. **Figure 4I** shows the comparative levels of the most commonly used biomarkers in the clusters, indicating significant differences and suggesting that suitable vaccination candidates may be identified by the presence of these biomarkers.

### Predicted responses to ICI therapy in the three clusters

We used the TIDE online tool for predicting the response to immunotherapy in the three clusters in the TCGA-BRCA and METABRIC cohorts and found that the tumor immune dysfunction scores for patients in Cluster B were significantly higher (**Supplementary Figure S4**), indicating that they may be less sensitive to immune checkpoint blockade.

### Relationships between the immune clusters and the tumor mutation burden

**Figure 5:**
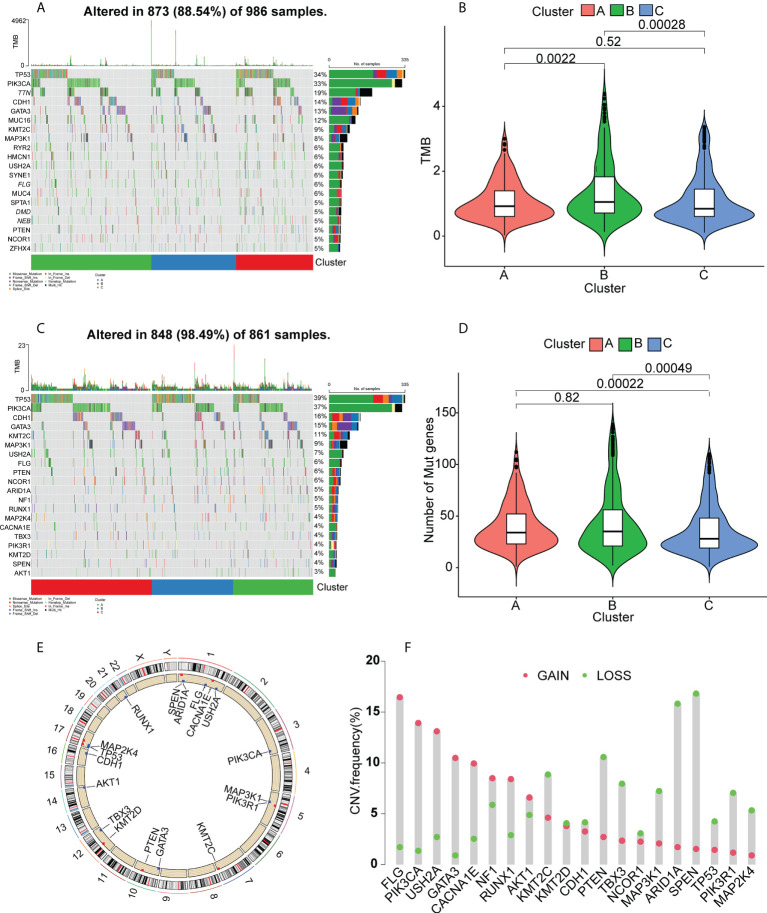
Gene mutation landscapes in the clusters. **(A)** Top 20 mutated genes in all clusters. **(B)** Tumor mutation burdens. **(C)** Top 20 mutated tumor-driver genes. **(D)** Tumor mutation burdens. **(E)** Chromosomal localizations of mutated tumor-driver genes. **(F)** CNV changes in mutated tumor-driver genes. Red dots represent increased CNV; green dots represent decreased CNV.

It has been found that patients with high tumor mutation burdens (TMBs) may respond well to immunotherapies due to the presence of numerous neoantigens (39). Elevated TMB values and somatic mutation rates have been linked with increased anti-tumor responses (40). Here, the TMB and numbers of mutated genes were determined for individual patients using the mutect2- processed TCGA mutation dataset in relation to the immune subtypes. The waterfall plot (**Figure 5A**) shows the 20 most commonly mutated immune-associated genes (**Figure 5A**). Both the TMB (**Figure 5B**) and the mutated gene numbers (**Figure 5D**) differed significantly among the three clusters, with Cluster B showing significantly elevated TMB values in comparison with Clusters A and C. This suggests that high TMB is linked to vaccination suitability. Subsequently, a set of tumor-driver genes for BC was extracted from the NCG database, and we evaluated the top 20 tumor-driver genes with the highest mutation frequencies in the three clusters and determined their chromosomal locations (**Figures 5C, E**). Copy number variations were raised in FLG, PIK3CA, USH2A, GATA3, CACNA1E, NF1, RUNX1, and AKT1, while reduced copy number variations were seen in KMT2C, KMT2D, CDH1, PTEN, TBX3, NCOR1, MAP3K1, ARID1A, SPEN, TP53, PIK3R1, and MAP2K4 (**Figure 5F**).

### Validation of potential immune subtypes and properties of BC

**Figure 6:**
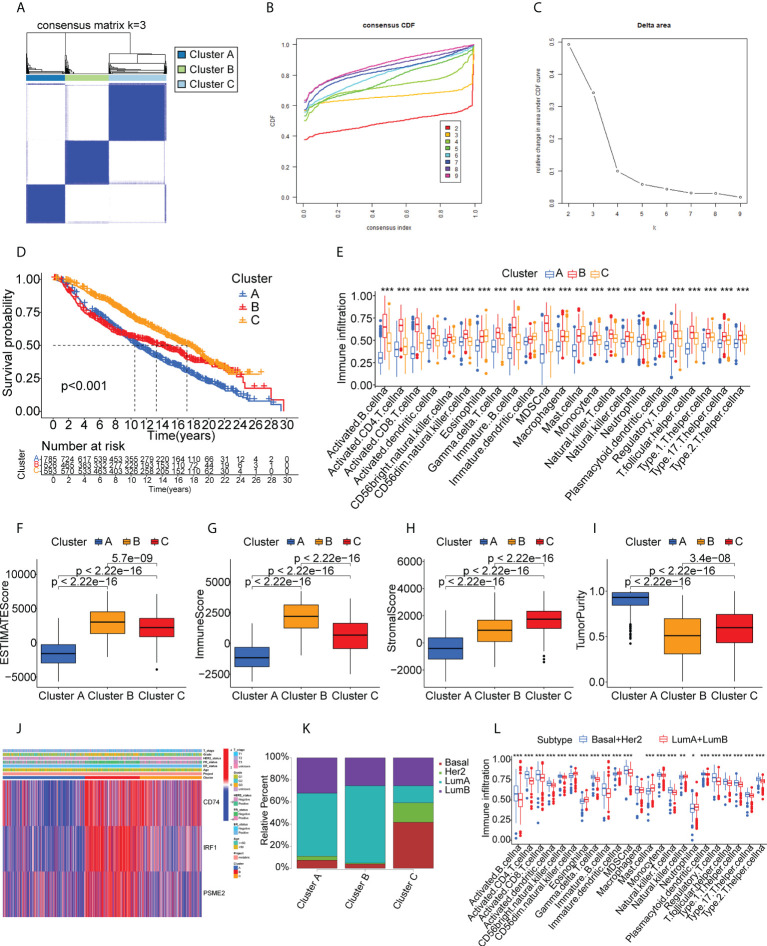
Cluster validation in the METABRIC cohort. **(A-C)** Cluster division based on levels of prognostic immune-related genes. **(D)** Survival analysis. **(E)** Boxplot showing immune cell infiltration, shown by ssGSEA enrichment. **(F-I)** Immune-stromal scores and tumor purity in the clusters, shown by ESTIMATE. **(J)** Heatmap of tumor antigen expression and clinical parameters. **(K)** Distribution of immune clusters in patients with different subtypes. **(L)** Boxplot of immune cell infiltration levels, determined by ssGSEA. "*" represents that p-value <0.05; "**" represents that p-value <0.01, and "***" represents that p-value <0.001.

We performed validation in the METABRIC cohort, constructing a consensus cluster in the same manner as the analysis in the TCGA cohort. The classifier model was most stable when k=3 (**Figures 6A–C**, **Supplementary Figure S5**). Information on patient classifications and clinical characteristics in the three clusters is also summarized in **Supplementary Tables S7** and **S8**. After t-SNE analysis to reduce the dimensionality of the data, it was found that the three clusters differed significantly (**Supplementary Figure S6**). At this point, the intra-cluster variance was the smallest and the inter-cluster variance was the largest. Cluster B was also linked to a better prognosis (**Figure 6D**). Twenty-eight immune characteristics were identified in the METABRIC cohort by ssGSEA, showing higher levels of immune infiltration in Cluster B (**Figure 6E**). The ESTIMATE immune and stromal scores for the clusters in the METABRIC cohort are shown in **Figure 6F–I**, indicating higher scores and lower tumor purity in Cluster B and thus a higher degree of immune cell infiltration. Interestingly, CD74, IRF1, and PSME2 were also strongly expressed in Cluster B (**Figure 6J**). This strongly suggested that Cluster B was more suitable for tumor mRNA vaccination. The distribution of the BC pathological subtypes in the three clusters also revealed that both the basal-like and HER2-overexpressing subtypes were more strongly represented in Cluster B (**Figure 6K**). Finally, the identification of the 28 immune signatures of the different subtypes in the TCGA cohort by the ssGSEA approach indicated that both the basal-like and HER2-overexpressing subtypes had relatively high numbers of immune cells (**Figure 6L**). In summary, the immunophenotyping was confirmed. Cluster B still had an active immune status and high TAA levels and was more suitable for mRNA vaccination targeting CD74, IRF1, PSME2. Furthermore, we suggest that basal-like and HER2-overexpressing BC patients may be more suited for mRNA vaccine therapy.

### Identification of a co-expression module of immune genes and prognostic biomarker in BC

**Figure 7:**
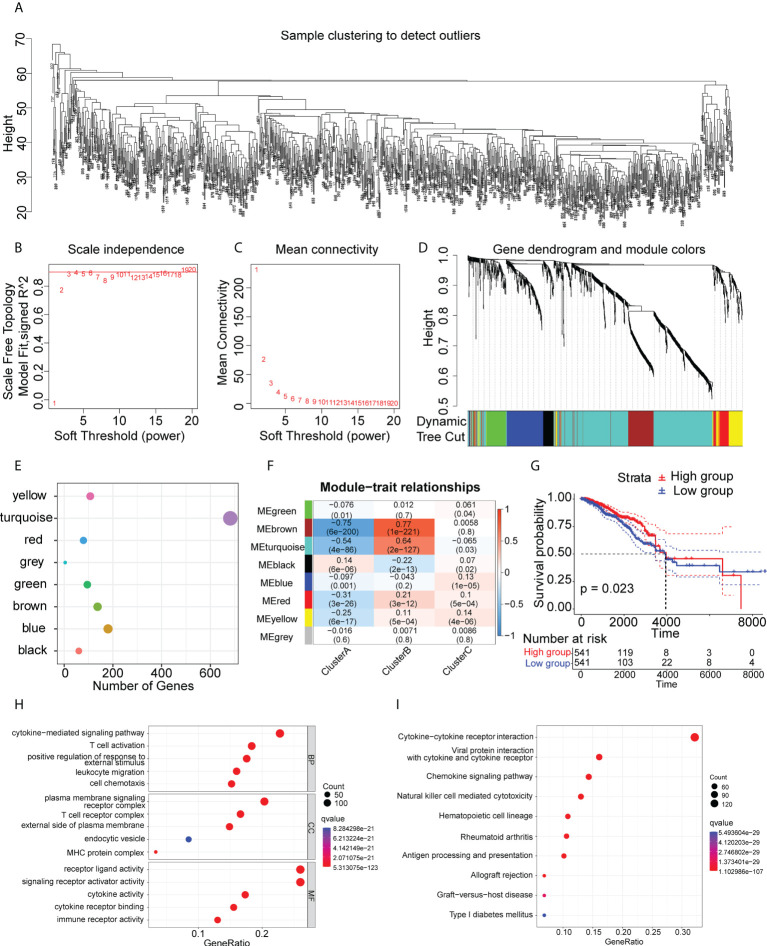
Immune gene co-expression modules. **(A-D)** Co-expression network of immune-associated genes. **(E)** Dot plot of co-expression modules. **(F)** Correlations between modules and immune clusters. **(G)** Survival analysis of patients with high and low scores for genes in the “turquoise” module. **(H)** GO analysis of genes in the “turquoise” module. **(I)** KEGG analysis of genes in the “turquoise” module.

Immune-associated genes were clustered using the WGCNA algorithm using the scale-free fit index and average connectivity and “three” selected as the soft threshold power (**Figure 7A–C**). The representation matrix was converted to an adjacent and then to a topological matrix. At least 30 genes were included in each network using average-linkage hierarchy clustering and the hybrid dynamic shear tree standard. After computation of the eigengenes for each module, the close modules were incorporated into a new module (deep split = 4 and min module size = 30) (**Figure 7D**). This led to the identification of eight modules in all immune-associated genes, with the gene numbers per module shown in **Figure 7E**. Examination of the associations between the modules and phenotypes showed a significant association between the “turquoise” module and Cluster B (suitable for vaccination) (**Figure 7F**). In addition, patients with higher scores of genes in the “turquoise” module survived longer than those with lower scores in the TCGA cohort (**Figure 7G**). GO and KEGG analyses showed that the genes in the turquoise module were strongly linked with immune activation and molecular processes of inflammation (**Figures 7H, I**). Further, we investigated the ability of all the genes for predicting prognosis in the Cluster B population (**Supplementary Table S9**). Finally, we identified five genes (ABCG4, MYO1E, REXO2, USP41, and VTA1P1) that showed good predictive power, having AUC values larger than 0.75 at one and three years, and greater than 0.70 at five years (**Supplementary Figure S7**) suggesting that these genes could be used as biomarkers after mRNA vaccination.

### Association of immune clusters with anti-cancer drug sensitivity

**Figure 8:**
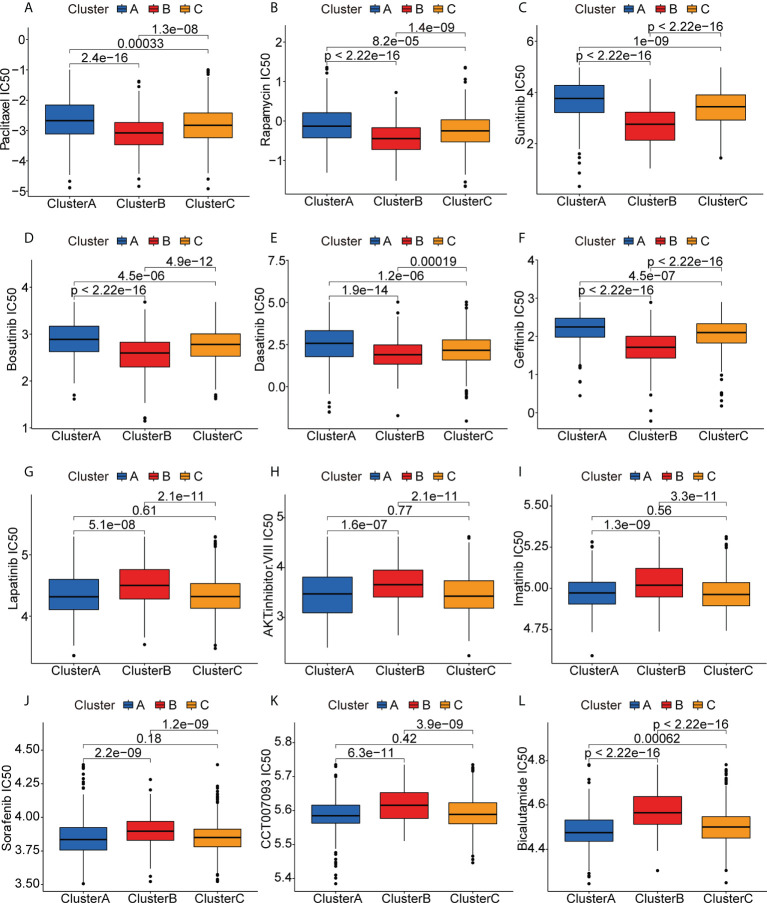
Anticancer drug sensitivities of patients in the different clusters. **(A-L)** IC50 values of anticancer drugs.

To examine the potential efficacies of treatments for patients in clusters that are unsuited for mRNA vaccination, we evaluated the sensitivities of patients in the different clusters to various chemotherapy drugs. Interestingly, we found that the patients in the Cluster B had lower IC50 values for paclitaxel, rapamycin, sunitinib, bosutinib, dasatinib, and gefitinib (**Figures 8A–E**), while the IC50 values of drugs such as lapatinib, AKT inhibitor VIII, imatinib, sorafenib, CCT007093, and bicalutamide were significantly lower in patients in Clusters A and C (**Figures 8F–L**). These differences in drug sensitivity between the clusters are reflections of tumor heterogeneity and suggest the importance of individualized treatment.

## Discussion

Over the past few decades, major technological innovations have enabled the use of mRNA as a more feasible vaccine candidate. For example, various modifications of the mRNA backbone and untranslated regions make the mRNA less sensitive to RNases, more stable, and highly translatable (41). In addition, optimization of the mRNA delivery systems has enhanced the ability of the vaccines to initiate an effective immune response (41). These factors, together with the progress in scale-up production, suggest that mRNA vaccines have significant advantages. Currently, there are numerous preclinical and clinical trials on mRNA cancer vaccines and the results show impressive efficacy (42–44). However, the clinical translation process is still limited by difficulties in antigen prediction and poor immunogenicity. One of the major obstacles to the development of effective cancer vaccines remains the difficulty of antigen selection (41).

Several features are required in an ideal antigen. The first of these is immunogenicity, that is, it needs to be capable of eliciting responses by T and B cells. This requires the processing and presentation of the antigen by major histocompatibility complex (MHC; also known as human leukocyte antigen, HLA, in humans) molecules on the cell surface (45). TAAs should also be specific to the tumor and present in significant amounts within the tumor (39). Of course, the dimensionality and variability of tumor antigens need to be taken into account.

Based on these properties, we first used BC gene expression profiles to identify potential antigens for mRNA vaccine formulation. We developed BC overexpression and mutation profiles and identified three promising mRNA vaccine candidates, namely CD74, IRF1, and PSME2. Elevated expression of these genes was not only associated with poor prognosis but also with greater infiltration of antigen-presenting cells and B cells. Immune subtype analysis showed the presence of a specific patient group that would be most likely to benefit from vaccination. This group showed a high degree of immune cell infiltration, together with elevated expression of ICPs and ICD modulators, as well as high TMBs. WGCNA identification of the characteristic gene modules in this group showed that ABCG4, MYO1E, REXO2, USP41, and VTA1P1 were useful biomarkers that could be used to assess vaccination response. Drug susceptibility analysis in patient populations unsuited for vaccination indicated the importance of individualized therapy. While further verification of these vaccine candidates is required, our findings on their potential for vaccine development are substantiated by previous studies.

CD74 has a dual role both as a part of the MHCII-like antigen presentation pathway and as a homologous receptor for macrophage migration inhibitory factor (MIF) (46). CD74 levels have been found to be linked with improved prognosis in invasive basal-like BC, possibly due to raised MHCII levels in tumor cells and greater numbers of tumor-infiltrating lymphocytes (47). CD74 knockdown has been found to reduce BC cell proliferation and increase apoptosis (48). Here, positive correlations were observed between CD74 and antigen-presenting cell infiltration, and single-cell sequencing analysis revealed that CD74 is highly expressed mainly in B cells, monocytes/macrophages, and pDCs of BC. Other studies have also found CD74 is expressed in BC circulating tumor cells (49). These findings suggest that CD74 may agitate immune cells surrounding BC to achieve anti-tumor responses, indicating its potential as a TAA vaccine candidate. Interferon regulatory factor (IRF)-1 is a transcription factor involved in innate and adaptive immunity. Deletions of the IRF1 locus at 5q31.1 has been observed in 50% of BRCA1-positive tumors (50). Surprisingly, while IRF-1 causes growth inhibition and cell death in BC cells by down-regulating molecules involved in the NF-κB pathway, the same effect was not observed in non-malignant human breast cells, reflecting the specificity of IRF1 (51). Moreover, IRF-1 binds to TNF-related apoptosis-inducing ligand (TRAIL) promoter, enhancing TRAIL-mediated killing of cancer cells. This action is not seen in normal cells, which ensures the safety of the vaccine to a certain extent (52). The IRF1/autophagy-related gene−7 (ATG7) signaling pathway is also key to tamoxifen resistance in estrogen receptor-positive (ER+) breast tumors (53). Combining endocrine therapy with compounds that effectively induce IRF1 expression *in vitro* is effective in reducing resistance to endocrine therapy in ER+ BCs (54). Of note, our single-cell sequencing analysis revealed that IRF1 was highly expressed mainly on endothelial cells, myoepithelial cells, and cancer-associated fibroblasts, while relatively low expression was observed in cancer cells and peripheral immune cells. Therefore, subsequent experimental studies are still necessary to determine whether IRF1 can be used as an mRNA vaccine. The PSME2 gene encodes Proteasome Activator Subunit 2 (PA28β), which is a subunit of PA28. PA28β expression is low in immature DCs and is strongly increased in mature DCs. Induction of PA28β expression not only enhances proteasome activity in activated DCs but also activates proteases that generate antigenic peptides presented by MHC class I molecules (55). And the result of our single-cell sequencing analysis supported the above conclusion that PSME2 was highly expressed in conventional dendritic cells of BC. This suggests that PSME2 may be involved in antigen processing and presentation in the BC tumor microenvironment. PSME2, although little studied in BC, has been found to have anticancer effects in a variety of cancers. PSME2 inhibits the growth, proliferation, and malignancy of esophageal squamous cell carcinoma cells and is considered a potential tumor suppressor (56). Knockdown of PSME2 is involved in the invasion and metastasis of gastric adenocarcinoma through upregulation of the chloride intracellular channel 1 (CLIC1) (57).

Our findings suggest new directions for the development of mRNA vaccines against BC, CD74, IRF1, and PSME2 were found to be promising antigens, and patients in the immune Cluster B were found to be better suited for vaccination. Nevertheless, there are still some limitations. For one, this work was based on retrospective data from publically available databases, and may thus be subject to selection bias. Furthermore, whether the expression of the identified potential antigen in tumor infiltrating immune cells has an impact on the efficacy of the vaccine still needs to be verified by subsequent experiments.

## Data availability statement

The original contributions presented in the study are included in the article/**Supplementary Material**. Further inquiries can be directed to the corresponding author.

## Author contributions

All authors contributed to the study conception and design. Collection and assembly of data were performed by RL, WW, and XG. The first draft of the manuscript was written by RL and LS. Manuscript revision was performed by LY, and JL and all authors commented on previous versions of the manuscript. Interactive review was performed by RL, LY, WZ, and JL. All authors read and approved the final manuscript.

## Funding

This work was supported by grants from the Shanxi Provincial Health Commission (2020065) and Shanxi Province Science Foundation for Youths, China [202103021223445].

## Acknowledgments

We thank the TCGA and METABRIC databases for the availability of the data.

## Conflict of interest

The authors declare that the research was conducted in the absence of any commercial or financial relationships that could be construed as a potential conflict of interest.

## Publisher’s note

All claims expressed in this article are solely those of the authors and do not necessarily represent those of their affiliated organizations, or those of the publisher, the editors and the reviewers. Any product that may be evaluated in this article, or claim that may be made by its manufacturer, is not guaranteed or endorsed by the publisher.
