# Supplementary material for: Identification of tumor antigens and immune subtypes in breast cancer for mRNA vaccine development
Source: Front Oncol. 2022 Sep 26;12:973712. doi: 10.3389/fonc.2022.973712 (PMC9548593; doi:10.3389/fonc.2022.973712)
Supplement: Supplementary file 1 [file DataSheet_1.pdf]

##scRNA-seq Analysis

```
library(Seurat)
library(stringr)
library(dplyr)
library(Matrix)
# Read in `matrix.mtx`
counts <- readMM("Wu_etal_2021_allcells_raw_counts.mtx")
dim(counts)
# Read in `genes.tsv`
library(readr)
genes <- read_tsv("Wu_etal_2021_allcells_genes.tsv", col_names = FALSE)
gene_ids <- genes$X1
# Read in `barcodes.tsv`
cells <- read_tsv("Wu_etal_2021_allcells_barcodes.tsv", col_names = FALSE)
cells$group=str_sub(cells$X1,1,3)
#extract breast cancer patients
cells_sub=cells[cells$group=="CID",]
cell_ids <- cells$X1
# Create a sparse matrix for more efficient computation
counts <- as(counts, "dgCMatrix")
# Make the column names as the cell IDs and the row names as the gene IDs
rownames(counts) <- gene_ids
colnames(counts) <- cell_ids
head(counts)
count_brca=counts[,colnames(counts)%in%cells_sub$X1]
dim(count_brca)
pbmc <- CreateSeuratObject(counts = count_brca,
                           project = "BRCA")
save(pbmc,file = "BRCA.Rdata")
#Read cell annotation information
meta <- read.table("meta.txt",sep = "\t",header = T)
meta=meta[-1,]
rownames(meta)=meta$NAME
samepatient=intersect(rownames(meta),cells_sub$X1)
length(samepatient)
meta=meta[samepatient,]
pbmc_sub=pbmc[,samepatient]
identical(colnames(pbmc_sub),rownames(meta))
pbmc_sub$cell_type=meta$CellType
#filter cell
pbmc_sub[["percent.mt"]] <- PercentageFeatureSet(pbmc_sub, pattern = "^MT-")
hist(pbmc_sub[["percent.mt"]]$percent.mt)
pbmc_sub<- subset(pbmc_sub, subset = nFeature_RNA > 200 & nFeature_RNA < 9000 &
percent.mt < 20)
VlnPlot(pbmc_sub, features = c("nFeature_RNA", "nCount_RNA", "percent.mt"), ncol = 3)
pbmc_sub <- NormalizeData(pbmc_sub, normalization.method = "LogNormalize", scale.factor =
10000)
pbmc_sub <- FindVariableFeatures(pbmc_sub, selection.method = "vst", nfeatures = 2000)
pbmc_sub <- ScaleData(pbmc_sub,vars.to.regress = c("percent.mt"))
pbmc_sub
###perform linear dimensional reduction###
```

```
pbmc_sub <- RunPCA(pbmc_sub, features = VariableFeatures(object = pbmc_sub))
####cluster the cells###
pbmc_sub <- FindNeighbors(pbmc_sub, dims = 1:30)
####Run non-linear dimensional reduction (UMAP)###
pbmc_sub <- RunUMAP(pbmc_sub, dims = 1:30)
pbmc_sub <- FindClusters(pbmc_sub, resolution = 0.6)###
DimPlot(pbmc_sub, reduction = "umap",label = T)
save(pbmc_sub,file = "BRCA_sub.Rdata")
load("BRCA_sub.Rdata")
Idents(pbmc_sub)=pbmc_sub$cell_type
#Visualization of target gene expression
library(ggsci)
cors<-pal_igv()(19)
features = c("CD74","IRF1","PSME2")
VlnPlot(pbmc_sub,features = features,cols = cors,pt.size = 0 )
DotPlot(pbmc_sub,features = features,cols = c("blue","red"))
```

#Gene Ontology (GO) analysis

```
library("clusterProfiler")
library("org.Hs.eg.db")
library("enrichplot")
library("ggplot2")
pvalueFilter=0.05
qvalueFilter=0.05
colorSel="qvalue"
if(qvalueFilter>0.05){
	colorSel="pvalue"
}
setwd("")
rt=read.table("interGene.txt", header=F, sep="\t", check.names=F)
genes=unique(as.vector(rt[,1]))
entrezIDs=mget(genes, org.Hs.egSYMBOL2EG, ifnotfound=NA)
entrezIDs=as.character(entrezIDs)
gene=entrezIDs[entrezIDs!="NA"]
kk=enrichGO(gene=gene, OrgDb=org.Hs.eg.db, pvalueCutoff=1, qvalueCutoff=1, ont="all",
readable=T)
GO=as.data.frame(kk)
GO=GO[(GO$pvalue<pvalueFilter & GO$qvalue<qvalueFilter),]
write.table(GO, file="GO.txt", sep="\t", quote=F, row.names = F)
showNum=10
if(nrow(GO)<30){
	showNum=nrow(GO)
}
pdf(file="bubble.pdf", width=9, height=7)
bub=dotplot(kk, showCategory=showNum, orderBy="GeneRatio", label_format=30,
split="ONTOLOGY", color=colorSel) + facet_grid(ONTOLOGY~., scale='free')
print(bub)
dev.off()
```

#Kyoto Encyclopedia of Genes and Genomes (KEGG) analysis

```
library("clusterProfiler")
library("org.Hs.eg.db")
library("enrichplot")
library("ggplot2")
pvalueFilter=0.05
qvalueFilter=0.05
colorSel="qvalue"
if(qvalueFilter>0.05){
	colorSel="pvalue"
}
setwd("")
rt=read.table("interGene.txt", header=F, sep="\t", check.names=F)
genes=unique(as.vector(rt[,1]))
entrezIDs=mget(genes, org.Hs.egSYMBOL2EG, ifnotfound=NA)
entrezIDs=as.character(entrezIDs)
rt=data.frame(genes, entrezID=entrezIDs)
gene=entrezIDs[entrezIDs!="NA"]
kk <- enrichKEGG(gene=gene, organism="hsa", pvalueCutoff=1, qvalueCutoff=1)
KEGG=as.data.frame(kk)
KEGG$geneID=as.character(sapply(KEGG$geneID,function(x)paste(rt$genes[match(strsplit(x,"/
")[[1]],as.character(rt$entrezID))],collapse="/")))
KEGG=KEGG[(KEGG$pvalue<pvalueFilter & KEGG$qvalue<qvalueFilter),]
write.table(KEGG, file="KEGG.txt", sep="\t", quote=F, row.names = F)
showNum=30
if(nrow(KEGG)<showNum){
	showNum=nrow(KEGG)
}
pdf(file="bubble.pdf", width = 9, height = 7)
dotplot(kk, showCategory=showNum, orderBy="GeneRatio", label_format=30, color=colorSel)
dev.off()
```

#Identification of Immunophenotyping in BC Patients

```
library(ConsensusClusterPlus)
expFile=""
workDir=""
setwd(workDir)
data=read.table(expFile, header=T, sep="\t", check.names=F, row.names=1)
data=as.matrix(data)
maxK=9
results=ConsensusClusterPlus(data,
		maxK=maxK,
		reps=50,
		pItem=0.8,
		pFeature=1,
		title=workDir,
		clusterAlg="km",
		distance="euclidean",
		seed=123456,
		plot="png")
clusterNum=3
cluster=results[[clusterNum]][["consensusClass"]]
```

```
cluster=as.data.frame(cluster)
colnames(cluster)=c("Cluster")
letter=c("A","B","C","D","E","F","G")
uniqClu=levels(factor(cluster$Cluster))
cluster$Cluster=letter[match(cluster$Cluster, uniqClu)]
clusterOut=rbind(ID=colnames(cluster), cluster)
write.table(clusterOut, file="Cluster.txt", sep="\t", quote=F, col.names=F)
```

#T-distributed stochastic neighbor embedding (t-SNE) analysis

```
library(Rtsne)
library(ggplot2)
setwd("")
bioPCA=function(inputFile=null,tsneFile=null){
	rt=read.table(inputFile, header=T, sep="\t", check.names=F, row.names=1)
	data=rt[c(3:(ncol(rt)-2))]
	risk=rt[,"risk"]
	tsneOut=Rtsne(data, dims=2, perplexity=10, verbose=F, max_iter=500,check_duplicates=F)
	tsne=data.frame(tSNE1 = tsneOut$Y[,1], tSNE2 = tsneOut$Y[,2],risk=risk)
	pdf(file=tsneFile, height=4.5, width=5.5)
	p=ggplot(data = tsne, aes(tSNE1, tSNE2)) + geom_point(aes(color = risk)) +
		scale_colour_manual(name="Risk",  values =c("red", "blue"))+
	   theme_bw()+
	   theme(plot.margin=unit(rep(1.5,4),'lines'))+
	   theme(panel.grid.major = element_blank(), panel.grid.minor = element_blank())
	print(p)
	dev.off()
}
bioPCA(inputFile="input.txt",tsneFile="train.t-SNE.pdf")
```

#Survival analysis

```
library(survival)
library(survminer)
clusterFile="Cluster.txt"
cliFile="time.txt"
setwd("C:\\biowolf\\prgTME\\23.ClusterSur")
cluster=read.table(clusterFile, header=T, sep="\t", check.names=F, row.names=1)
rownames(cluster)=gsub("(.*?)\\_(.*?)", "\\2", rownames(cluster))
cli=read.table(cliFile, header=T, sep="\t", check.names=F, row.names=1)
colnames(cli)=c("futime", "fustat")
cli$futime=cli$futime/365
sameSample=intersect(row.names(cluster), row.names(cli))
rt=cbind(cli[sameSample,,drop=F], cluster[sameSample,,drop=F])
length=length(levels(factor(rt$Cluster)))
diff=survdiff(Surv(futime, fustat) ~ Cluster, data = rt)
pValue=1-pchisq(diff$chisq, df=length-1)
if(pValue<0.001){
	pValue="p<0.001"
}else{
	pValue=paste0("p=",sprintf("%.03f",pValue))
}
```

```
fit <- survfit(Surv(futime, fustat) ~ Cluster, data = rt)
print(surv_median(fit))
bioCol=c("#0066FF","#FF9900","#FF0000","#6E568C","#7CC767","#223D6C","#D20A13","#
FFD121","#088247","#11AA4D")
bioCol=bioCol[1:length]
surPlot=ggsurvplot(fit,
                   data=rt,
                   conf.int=F,
                   pval=pValue,
                   pval.size=6,
                   legend.title="Cluster",
                   legend.labs=levels(factor(rt[,"Cluster"])),
                   legend = c(0.8, 0.8),
                   font.legend=10,
                   xlab="Time(years)",
                   break.time.by = 1,
                   palette = bioCol,
                   surv.median.line = "hv",
                   risk.table=T,
                   cumevents=F,
                   risk.table.height=.25)
pdf(file="survival.pdf",onefile = FALSE,width=7,height=5.5)
print(surPlot)
dev.off()
```

#Heat map of the expression of tumor antigens between clusters

```
library(pheatmap)
expFile="prgGeneExp.txt"
clusterFile="Cluster.txt"
cliFile="clinical.txt"
setwd("")
exp=read.table(expFile, header=T, sep="\t", check.names=F, row.names=1)
exp=t(exp)
cluster=read.table(clusterFile, header=T, sep="\t", check.names=F, row.names=1)
sameSample=intersect(row.names(exp), row.names(cluster))
exp=exp[sameSample, , drop=F]
cluster=cluster[sameSample, , drop=F]
expCluster=cbind(exp, cluster)
Project=gsub("(.*?)\\_.*", "\\1", rownames(expCluster))
rownames(expCluster)=gsub("(.*?)\\_(.*?)", "\\2", rownames(expCluster))
expCluster=cbind(expCluster, Project)
cli=read.table(cliFile, header=T, sep="\t", check.names=F, row.names=1)
cli[,"Age"]=ifelse(cli[,"Age"]=="unknown", "unknown", ifelse(cli[,"Age"]>50,">50","<=50"))
sameSample=intersect(row.names(expCluster), row.names(cli))
expCluster=expCluster[sameSample,,drop=F]
cli=cli[sameSample,,drop=F]
data=cbind(expCluster, cli)
data=data[order(data$Cluster),]
Type=data[,((ncol(exp)+1):ncol(data))]
data=t(data[,1:ncol(exp)])
```

```
bioCol=c("#0066FF","#FF9900","#FF0000")
ann_colors=list()
prgCluCol=bioCol[1:length(levels(factor(Type$Cluster)))]
names(prgCluCol)=levels(factor(Type$Cluster))
ann_colors[["Cluster"]]=prgCluCol
pdf("heatmap.pdf", width=7.5, height=5)
pheatmap(data,
        annotation=Type,
        annotation_colors = ann_colors,
        color = colorRampPalette(c(rep("blue",5), "white", rep("red",5)))(100),
        cluster_cols =F,
        cluster_rows =F,
        scale="row",
        show_colnames=F,
        fontsize=6,
        fontsize_row=6,
        fontsize_col=6)
dev.off()
```

# GSVA of biological pathways between three distinct subtypes

```
library(limma)
library(GSEABase)
library(GSVA)
library(pheatmap)

expFile="merge.txt"
clusterFile="Cluster.txt"
gmtFile="c2.cp.kegg.v7.4.symbols.gmt"
setwd("")
rt=read.table(expFile, header=T, sep="\t", check.names=F)
rt=as.matrix(rt)
rownames(rt)=rt[,1]
exp=rt[,2:ncol(rt)]
dimnames=list(rownames(exp), colnames(exp))
data=matrix(as.numeric(as.matrix(exp)), nrow=nrow(exp), dimnames=dimnames)
data=avereps(data)
geneSets=getGmt(gmtFile, geneIdType=SymbolIdentifier())
gsvaResult=gsva(data,
              geneSets,
              min.sz=10,
              max.sz=500,
              verbose=TRUE,
              parallel.sz=1)
gsvaOut=rbind(id=colnames(gsvaResult), gsvaResult)
write.table(gsvaOut, file="gsvaOut.txt", sep="\t", quote=F, col.names=F)
cluster=read.table(clusterFile, header=T, sep="\t", check.names=F, row.names=1)
gsvaResult=t(gsvaResult)
sameSample=intersect(row.names(gsvaResult), row.names(cluster))
gsvaResult=gsvaResult[sameSample,,drop=F]
cluster=cluster[sameSample,,drop=F]
gsvaCluster=cbind(gsvaResult, cluster)
```

```
Project=gsub("(.*?)\\_.*", "\\1", rownames(gsvaCluster))
gsvaCluster=cbind(gsvaCluster, Project)
adj.P.Val.Filter=0.05
allType=as.vector(gsvaCluster$Cluster)
comp=combn(levels(factor(allType)), 2)
for(i in 1:ncol(comp)){
	treat=gsvaCluster[gsvaCluster$Cluster==comp[2,i],]
	con=gsvaCluster[gsvaCluster$Cluster==comp[1,i],]
	data=rbind(con, treat)
	Type=as.vector(data$Cluster)
	ann=data[,c(ncol(data), (ncol(data)-1))]
	data=t(data[,-c((ncol(data)-1), ncol(data))])
	design=model.matrix(~0+factor(Type))
	colnames(design)=levels(factor(Type))
	fit=lmFit(data, design)
	contrast=paste0(comp[2,i], "-", comp[1,i])
	cont.matrix=makeContrasts(contrast, levels=design)
	fit2=contrasts.fit(fit, cont.matrix)
	fit2=eBayes(fit2)
	allDiff=topTable(fit2,adjust='fdr',number=200000)
	allDiffOut=rbind(id=colnames(allDiff),allDiff)
	write.table(allDiffOut, file=paste0(contrast, ".all.txt"), sep="\t", quote=F, col.names=F)
	diffSig=allDiff[with(allDiff, (abs(logFC)>0.1 & adj.P.Val < adj.P.Val.Filter )), ]
	diffSigOut=rbind(id=colnames(diffSig),diffSig)
	write.table(diffSigOut, file=paste0(contrast, ".diff.txt"), sep="\t", quote=F, col.names=F)
	bioCol=c("#0066FF","#FF9900","#FF0000")
	ann_colors=list()
	m6aCluCol=bioCol[1:length(levels(factor(allType)))]
	names(m6aCluCol)=levels(factor(allType))
	ann_colors[["Cluster"]]=m6aCluCol[c(comp[1,i], comp[2,i])]
	termNum=8
	diffTermName=as.vector(rownames(diffSig))
	diffLength=length(diffTermName)
	if(diffLength<termNum){termNum=diffLength}
	hmGene=diffTermName[1:termNum]
	hmExp=data[hmGene,]
	pdf(file=paste0(contrast,".heatmap.pdf"), width=10, height=6)
	pheatmap(hmExp,
		annotation=ann,
		annotation_colors = ann_colors,
		color = colorRampPalette(c(rep("blue",2), "white", rep("red",2)))(50),
		cluster_cols =F,
		show_colnames = F,
		gaps_col=as.vector(cumsum(table(Type))),
		scale="row",
		fontsize = 8,
		fontsize_row=6,
		fontsize_col=8)
	dev.off()
}
```

#Heat map of immune cell infiltration levels assessed based on ssGSEA enrichment.

```
library(reshape2)
library(ggpubr)
library(limma)
library(GSEABase)
library(GSVA)
expFile="merge.txt"
clusterFile="Cluster.txt"
gmtFile="immune.gmt"
setwd("")
rt=read.table(expFile, header=T, sep="\t", check.names=F)
rt=as.matrix(rt)
rownames(rt)=rt[,1]
exp=rt[,2:ncol(rt)]
dimnames=list(rownames(exp),colnames(exp))
data=matrix(as.numeric(as.matrix(exp)),nrow=nrow(exp),dimnames=dimnames)
data=avereps(data)
geneSets=getGmt(gmtFile, geneIdType=SymbolIdentifier())
ssgseaScore=gsva(data, geneSets, method='ssgsea', kcdf='Gaussian', abs.ranking=TRUE)
normalize=function(x){
  return((x-min(x))/(max(x)-min(x)))}
ssgseaScore=normalize(ssgseaScore)
ssgseaOut=rbind(id=colnames(ssgseaScore), ssgseaScore)
write.table(ssgseaOut,file="ssGSEA.result.txt",sep="\t",quote=F,col.names=F)
cluster=read.table(clusterFile, header=T, sep="\t", check.names=F, row.names=1)
ssgseaScore=t(ssgseaScore)
sameSample=intersect(row.names(ssgseaScore), row.names(cluster))
ssgseaScore=ssgseaScore[sameSample,,drop=F]
cluster=cluster[sameSample,,drop=F]
scoreCluster=cbind(ssgseaScore, cluster)
data=melt(scoreCluster, id.vars=c("Cluster"))
colnames(data)=c("Cluster", "Immune", "Fraction")
bioCol=c("#0066FF","#FF9900","#FF0000")
bioCol=bioCol[1:length(levels(factor(data[,"Cluster"])))]
p=ggboxplot(data, x="Immune", y="Fraction", color="Cluster",
     ylab="Immune infiltration",
     xlab="",
     legend.title="Cluster",
     palette=bioCol)
p=p+rotate_x_text(50)
pdf(file="boxplot.pdf", width=8, height=6.5)
p+stat_compare_means(aes(group=Cluster),symnum.args=list(cutpoints = c(0, 0.001, 0.01, 0.05, 1), symbols = c("***", "**", "*", "")),label = "p.signif")
dev.off()
```

#Comparison of immune stromal cell scores for each sample between different clusters by the "Estimate" algorithm

```
library(limma)
library(estimate)
inputFile="merge.txt"
setwd("C:\\biowolf\\prgTME\\46.estimate")
```

```
rt=read.table(inputFile, header=T, sep="\t", check.names=F)
rt=as.matrix(rt)
rownames(rt)=rt[,1]
exp=rt[,2:ncol(rt)]
dimnames=list(rownames(exp),colnames(exp))
data=matrix(as.numeric(as.matrix(exp)),nrow=nrow(exp),dimnames=dimnames)
data=avereps(data)
out=rbind(ID=colnames(data),data)
write.table(out,file="uniq.symbol.txt",sep="\t",quote=F,col.names=F)
filterCommonGenes(input.f="uniq.symbol.txt",
                  output.f="commonGenes.gct",
                  id="GeneSymbol")
estimateScore(input.ds = "commonGenes.gct",
              output.ds="estimateScore.gct")
scores=read.table("estimateScore.gct", skip=2, header=T)
rownames(scores)=scores[,1]
scores=t(scores[,3:ncol(scores)])
rownames(scores)=gsub("\\.", "\\-", rownames(scores))
out=rbind(ID=colnames(scores), scores)
write.table(out, file="TMEscores.txt", sep="\t", quote=F, col.names=F)
```

#Visualization of gene mutations between clusters by the "maftool" R package

```
library(maftools)
setwd("")
geneRT=read.table("gene.txt", header=T, sep="\t", check.names=F, row.names=1)
gene=row.names(geneRT)
pdf(file="oncoplot.pdf", width=8, height=7.5)
maf=read.maf(maf="input.maf")
oncoplot(maf=maf, genes=gene, fontSize=0.5, draw_titv=T)
dev.off()
```

#Mapping the location of genes on chromosomes

```
setwd("")
cytoBandIdeogram=read.table("refer.txt", header=T, sep="\t")
chr.exclude <- NULL
cyto.info <- cytoBandIdeogram
tracks.inside <- 5
tracks.outside <- 0
RCircos.Set.Core.Components(cyto.info, chr.exclude, tracks.inside, tracks.outside)
rcircos.params <- RCircos.Get.Plot.Parameters()
rcircos.params$text.size=0.8
rcircos.params$point.size=5
RCircos.Reset.Plot.Parameters(rcircos.params)
pdf(file="RCircos.pdf", width=8, height=8)
RCircos.Set.Plot.Area()
RCircos.Chromosome.Ideogram.Plot()
RCircos.Scatter.Data=read.table("Rcircos.scatter.txt", header=T, sep="\t", check.names=F)
data.col <- 4
track.num <- 1
side <- "in"
```

```
RCircos.Scatter.Plot(RCircos.Scatter.Data, data.col, track.num, side, by.fold=0.1)
RCircos.Gene.Label.Data=read.table("Rcircos.geneLabel.txt", header=T, sep="\t", check.names=F)
name.col <- 4
side <- "in"
track.num <- 2
RCircos.Gene.Connector.Plot(RCircos.Gene.Label.Data, track.num, side)
track.num <- 3
RCircos.Gene.Name.Plot(RCircos.Gene.Label.Data, name.col, track.num, side)
dev.off()
```

# Weighted Gene Co-expression Network Analysis

```
library(WGCNA)
setwd("")
inputdata1=""
data0=read.table(inputdata1,sep="\t",row.names=1,header=T,check.names=F,quote="!")
datSummary=rownames(data0)
datExpr = t(data0)
no.samples = dim(datExpr)[[1]]
dim(datExpr)
powers1=c(seq(1,10,by=1),seq(12,20,by=2))
RpowerTable=pickSoftThreshold(datExpr, powerVector=powers1)[[2]]
cex1=1
par(mfrow=c(1,2))
pdf("beta.pdf")
plot(RpowerTable[,1], -sign(RpowerTable[,3])*RpowerTable[,2],xlab="
Soft Threshold (power)",ylab="Scale Free Topology Model Fit,signed R^2",type="n")
text(RpowerTable[,1], -sign(RpowerTable[,3])*RpowerTable[,2], labels=powers1,cex=cex1,col="red")
abline(h=0.85,col="red")
plot(RpowerTable[,1], RpowerTable[,5],xlab="Soft Threshold (power)",ylab="Mean Connectivity", type="n")
text(RpowerTable[,1], RpowerTable[,5], labels=powers1, cex=cex1,col="red")
dev.off()
beta1=4
Connectivity=softConnectivity(datExpr,power=beta1)
pdf("scalefree.pdf",15,10)
par(mfrow=c(1,1))
scaleFreePlot(Connectivity, main=paste("soft threshold, power=",beta1), truncated=T)
dev.off()
ConnectivityCut = 1000
ConnectivityRank = rank(-Connectivity)
restConnectivity = ConnectivityRank <= ConnectivityCut
ADJrest = adjacency(datExpr[,restConnectivity], power=beta1)
dissTOM=TOMdist(ADJrest)
hierTOM = hclust(as.dist(dissTOM),method="average")
colorh1= cutreeStaticColor(hierTOM,cutHeight = 0.8, minSize = 3)
pdf("module.pdf")
par(mfrow=c(2,1),mar=c(2,4,1,1))
```

```
plot(hierTOM, main="Cluster Dendrogram", labels=F, xlab="", sub="")
plotColorUnderTree(hierTOM,colors=data.frame(module=colorh1))
title("Module (branch) color")
dev.off()
pdf("TOM.pdf")
TOMplot(dissTOM , hierTOM, colorh1, terrainColors=TRUE)
dev.off()
pdf("cmd.pdf")
cmd1=cmdscale(as.dist(dissTOM),3)
pairs(cmd1, col=as.character(colorh1),  main="MDS plot")
dev.off()
library(scatterplot3d)
pdf("3d.pdf")
par(mfrow=c(1,1), mar=c(4,3,2,3)+0.1)
scatterplot3d(cmd1,color=colorh1,angle=250,
xlab="Scaling Axis 1", ylab="Scaling Axis 2", zlab="Scaling Axis 3")
dev.off()
datME=moduleEigengenes(datExpr[,restConnectivity],colorh1)[[1]]
dissimME=1-(t(cor(datME, method="p")))/2
hclustdatME=hclust(dist(dissimME), method="average" )
pdf("modul_cluster.pdf")
par(mfrow=c(1,1))
plot(hclustdatME, main="Clustering tree based on the module eigengenes of modules")
dev.off()
pdf("modul_cor.pdf")
pairs(datME)
dev.off()
modul<-signif(cor(datME, use="p"), 2)
write.table(modul,"modul_cor.txt",sep="\t",quote=F)
datME=moduleEigengenes(datExpr,colorh1)[[1]]
color1=rep("grey",dim(datExpr)[[2]])
color1=as.character(colorh1)
datKME=signedKME(datExpr, datME)
datout=data.frame(datSummary, colorNEW=color1,datKME )
write.table(datout, "gene_module.xls", sep="\t", row.names=F,quote=F)
exportNetworkToCytoscape(ADJrest,edgeFile="edge.txt",nodeFile="node.txt",threshold = 0.5)
inputclinial="Cluster.txt"
dataclinial = read.table(inputclinial,sep="\t",row.names=1,header=T,check.names=F,quote="!")
nGenes = ncol(datExpr)
nSamples = nrow(datExpr)
MEs0 = moduleEigengenes(datExpr,colorh1)$eigengenes
MEsFemale = orderMEs(MEs0)
modul_clinical_cor = cor(MEsFemale, dataclinial, use = "p")
write.table(modul_clinical_cor,"module-clinial-cor.xls",sep="\t",quote=F)
modul_clinical_p = corPvalueStudent(modul_clinical_cor, nSamples)
write.table(modul_clinical_p,"modul-clinical-p.xls",sep="\t",quote=F)
textMatrix = paste(signif(modul_clinical_cor, 2), " (", signif(modul_clinical_p, 1), ")",sep = "")
dim(textMatrix) = dim(modul_clinical_cor)
pdf("modul-clinical.pdf")
par(mar = c(6, 8.5, 3, 3))
labeledHeatmap(Matrix = modul_clinical_cor, xLabels = names(dataclinial),
```

```
  yLabels = names(MEsFemale),ySymbols = names(MEsFemale), colorLabels = FALSE,
  colors = greenWhiteRed(50), textMatrix = textMatrix, setStdMargins = FALSE,
  cex.text = 1, zlim = c(-1,1), main = paste("Module-trait relationships"))
dev.off()
```

#Anticancer Drug Sensitivity Analyses between clusters

```
library(limma)
library(ggpubr)
library(pRRophetic)
library(ggplot2)
pFilter=0.001
expFile="merge.txt"
riskFile="Cluster.txt"
setwd("")
allDrugs=c("A.443654", "A.770041", "ABT.263", "ABT.888", "AG.014699", "AICAR",
"AKT.inhibitor.VIII", "AMG.706", "AP.24534", "AS601245", "ATRA", "AUY922", "Axitinib",
"AZ628", "AZD.0530", "AZD.2281", "AZD6244", "AZD6482", "AZD7762", "AZD8055",
"BAY.61.3606", "Bexarotene", "BI.2536", "BIBW2992", "Bicalutamide", "BI.D1870",
"BIRB.0796", "Bleomycin", "BMS.509744", "BMS.536924", "BMS.708163", "BMS.754807",
"Bortezomib", "Bosutinib", "Bryostatin.1", "BX.795", "Camptothecin", "CCT007093",
"CCT018159", "CEP.701", "CGP.082996", "CGP.60474", "CHIR.99021", "CI.1040", "Cisplatin",
"CMK", "Cyclopamine", "Cytarabine", "Dasatinib", "DMOG", "Docetaxel", "Doxorubicin",
"EHT.1864", "Elesclomol", "Embelin", "Epothilone.B", "Erlotinib", "Etoposide", "FH535",
"FTI.277", "GDC.0449", "GDC0941", "Gefitinib", "Gemcitabine", "GNF.2", "GSK269962A",
"GSK.650394", "GW.441756", "GW843682X", "Imatinib", "IPA.3", "JNJ.26854165", "JNK.9L",
"JNK.Inhibitor.VIII", "JW.7.52.1", "KIN001.135", "KU.55933", "Lapatinib", "Lenalidomide",
"LFM.A13", "Metformin", "Methotrexate", "MG.132", "Midostaurin", "Mitomycin.C",
"MK.2206", "MS.275", "Nilotinib", "NSC.87877", "NU.7441", "Nutlin.3a", "NVP.BEZ235",
"NVP.TAE684", "Obatoclax.Mesylate", "OSI.906", "PAC.1", "Paclitaxel", "Parthenolide",
"Pazopanib", "PD.0325901", "PD.0332991", "PD.173074", "PF.02341066", "PF.4708671",
"PF.562271", "PHA.665752", "PLX4720", "Pyrimethamine", "QS11", "Rapamycin", "RDEA119",
"RO.3306", "Roscovitine", "Salubrinal", "SB.216763", "SB590885", "Shikonin", "SL.0101.1",
"Sorafenib", "S.Trityl.L.cysteine", "Sunitinib", "Temsirolimus", "Thapsigargin", "Tipifarnib",
"TW.37", "Vinblastine", "Vinorelbine", "Vorinostat", "VX.680", "VX.702", "WH.4.023",
"WO2009093972", "WZ.1.84", "X17.AAG", "X681640", "XMD8.85", "Z.LLNle.CHO",
"ZM.447439")
rt = read.table(expFile, header=T, sep="\t", check.names=F)
rt=as.matrix(rt)
rownames(rt)=rt[,1]
exp=rt[,2:ncol(rt)]
dimnames=list(rownames(exp),colnames(exp))
data=matrix(as.numeric(as.matrix(exp)),nrow=nrow(exp),dimnames=dimnames)
data=avereps(data)
data=data[rowMeans(data)>0,]
colnames(data)=gsub("(.*?)\\_(.*?)", "\\2", colnames(data))
ClusterRT=read.table(riskFile, header=T, sep="\t", check.names=F, row.names=1)
for(drug in allDrugs){
	senstivity=pRRopheticPredict(data, drug, selection=1)
	senstivity=senstivity[senstivity!="NaN"]
	senstivity[senstivity>quantile(senstivity,0.99)]=quantile(senstivity,0.99)
```

```
	sameSample=intersect(row.names(ClusterRT), names(senstivity))
	Cluster=ClusterRT[sameSample, "Cluster",drop=F]
	senstivity=senstivity[sameSample]
	rt=cbind(Cluster, senstivity)
	rt$Cluster=factor(rt$Cluster, levels=c("A", "B","C"))
	type=levels(factor(rt[,"Cluster"]))
	comp=combn(type, 2)
	my_comparisons=list()
	for(i in 1:ncol(comp)){my_comparisons[[i]]<-comp[,i]}
	test=wilcox.test(senstivity~Cluster, data=rt)

	if(test$p.value<pFilter){
		boxplot=ggboxplot(rt, x="Cluster", y="senstivity", fill="Cluster",
		                  xlab="Cluster",
		                  ylab=paste0(drug, " senstivity (IC50)"),
		                  legend.title="Cluster",
		                  palette=c("#0066FF","#FF0000","#FF0000")
		                 )+
			stat_compare_means(comparisons=my_comparisons)
		pdf(file=paste0("durgSenstivity.", drug, ".pdf"), width=5, height=4.5)
		print(boxplot)
		dev.off()
	}
}
```
