# Supplementary figures and images for: Identification of tumor antigens and immune subtypes in breast cancer for mRNA vaccine development
Source: Front Oncol. 2022 Sep 26;12:973712. doi: 10.3389/fonc.2022.973712 (PMC9548593; doi:10.3389/fonc.2022.973712)

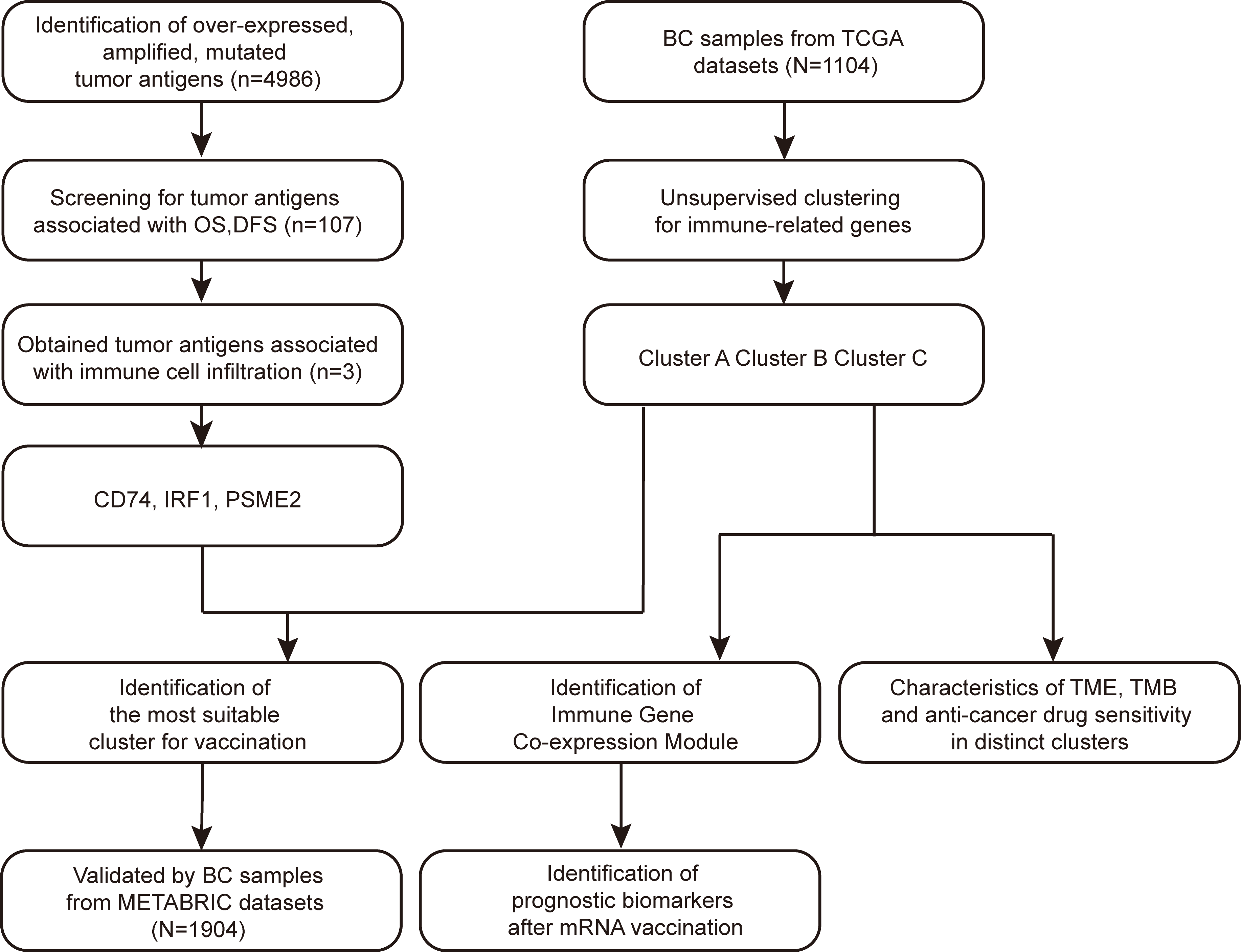

Supplement: Supplementary file 2 [file Image_1.tif]

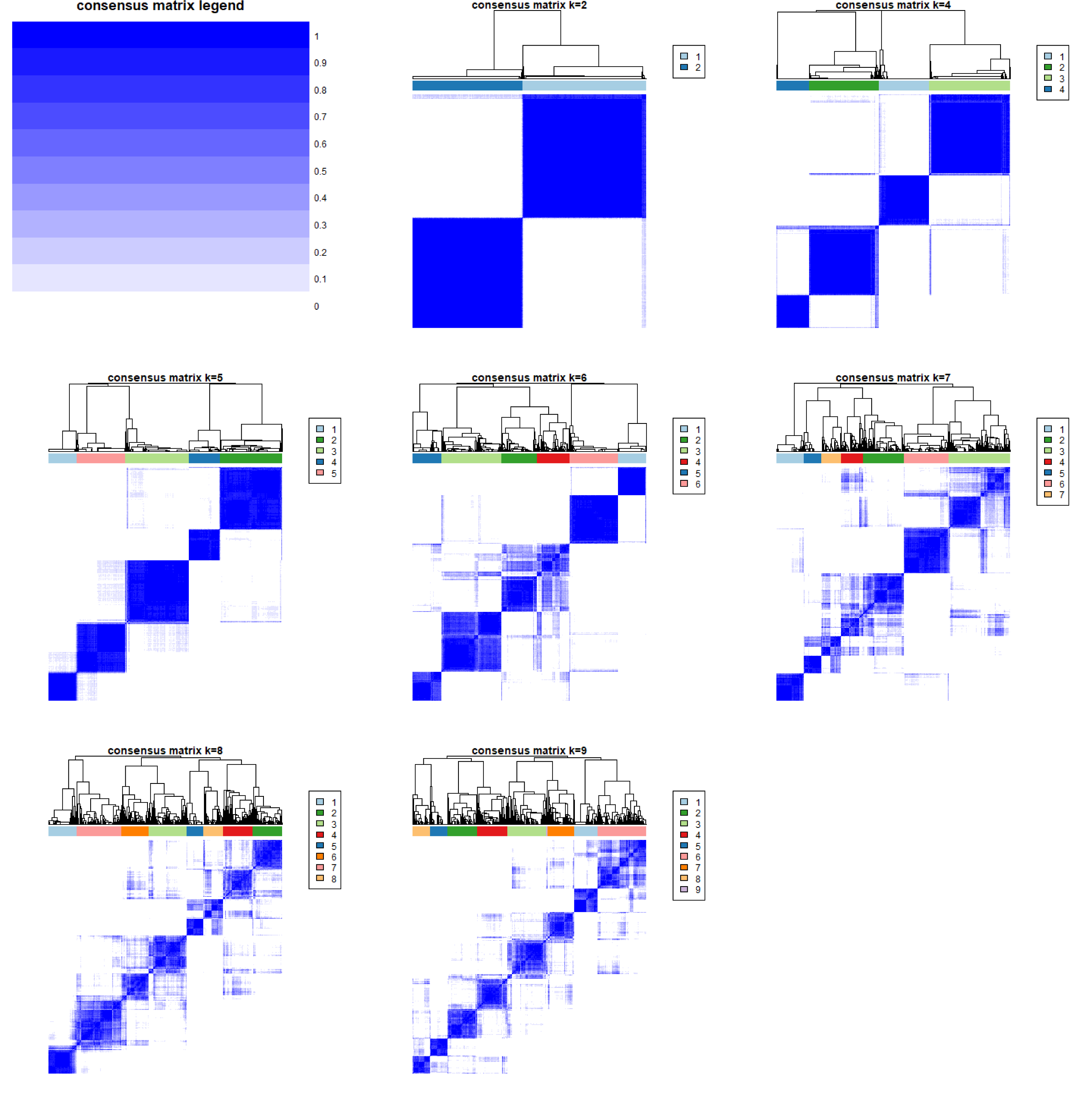

Supplement: Supplementary file 3 [file Image_2.tif]

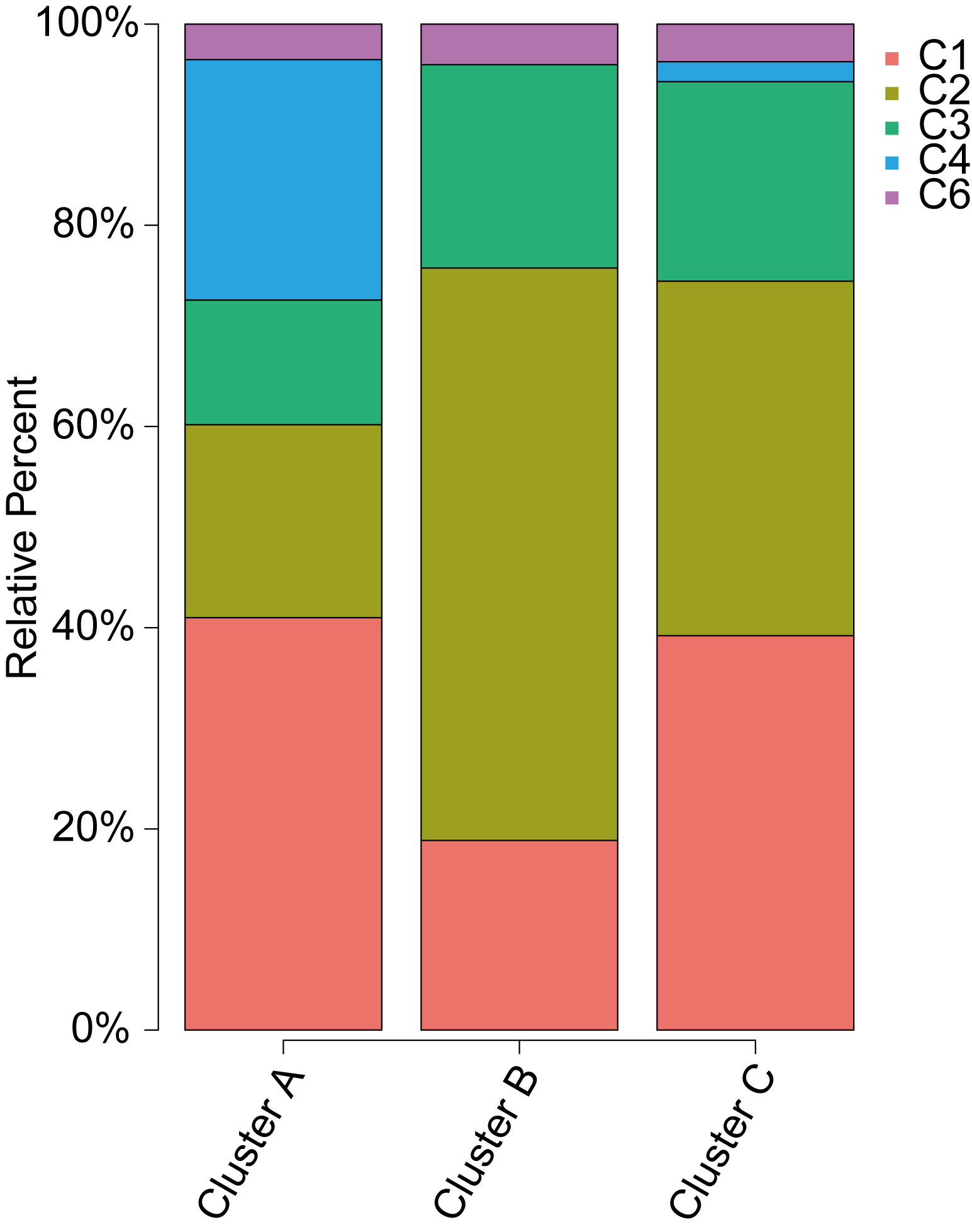

Supplement: Supplementary file 4 [file Image_3.tif]

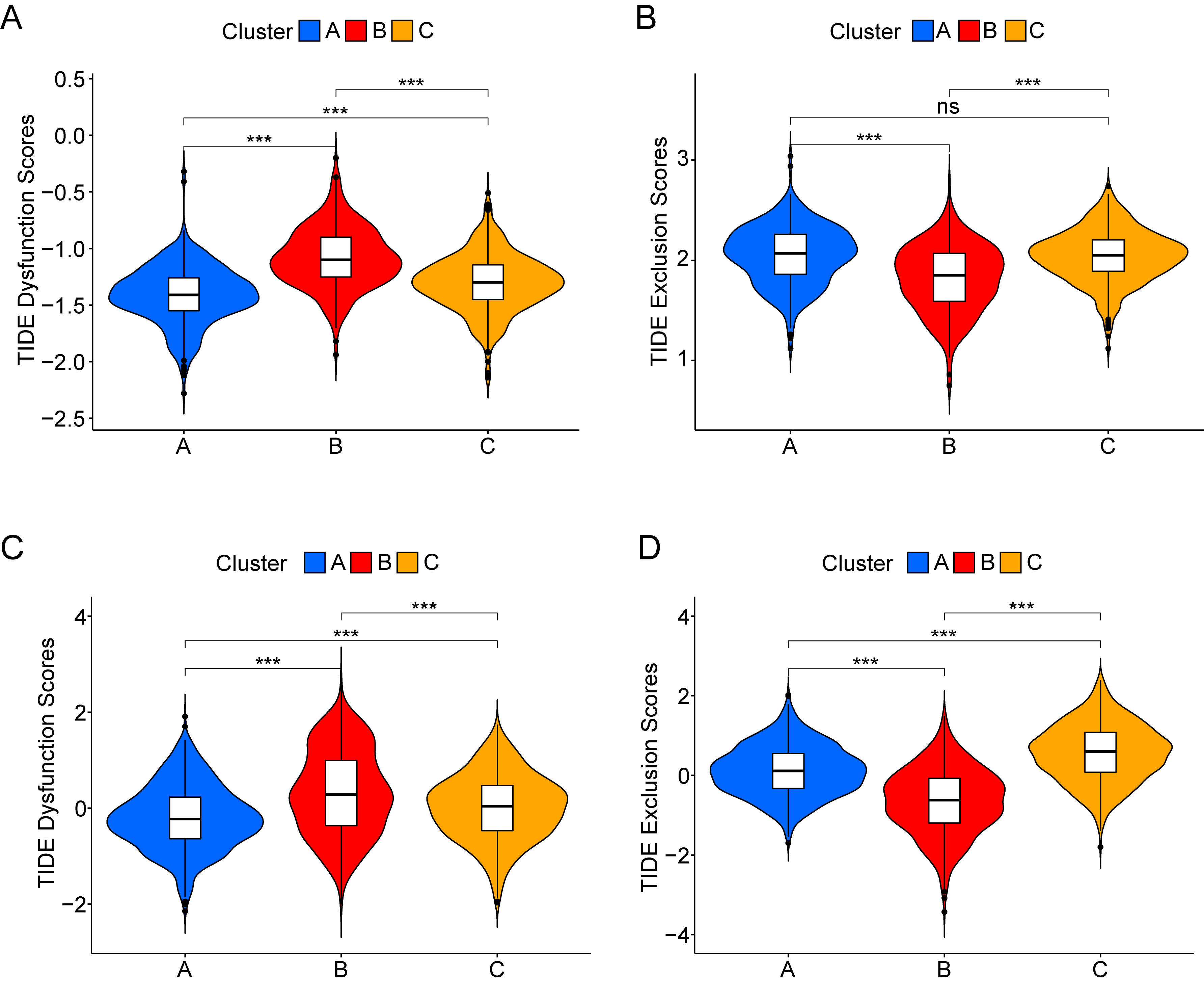

Supplement: Supplementary file 5 [file Image_4.tif]

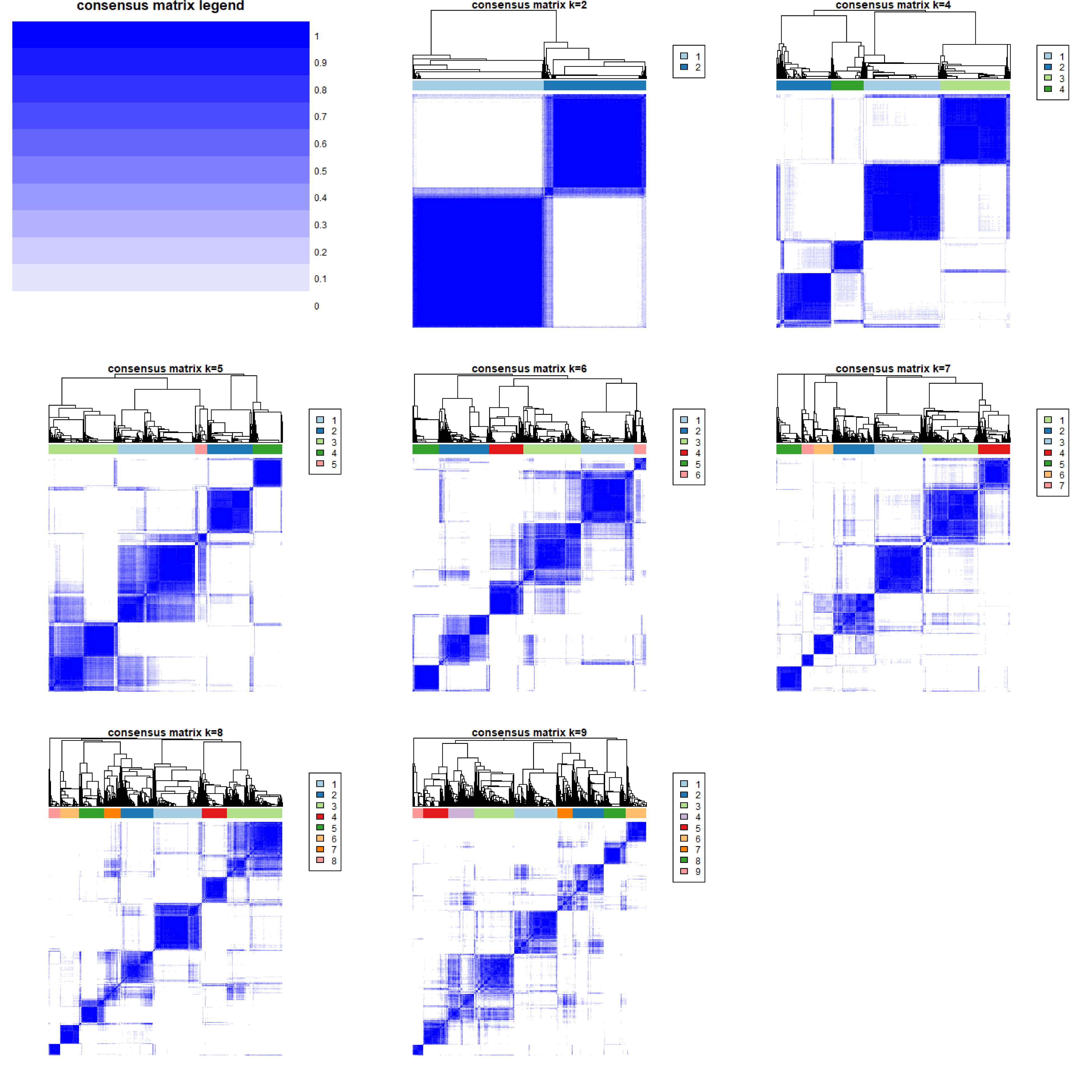

Supplement: Supplementary file 6 [file Image_5.tif]

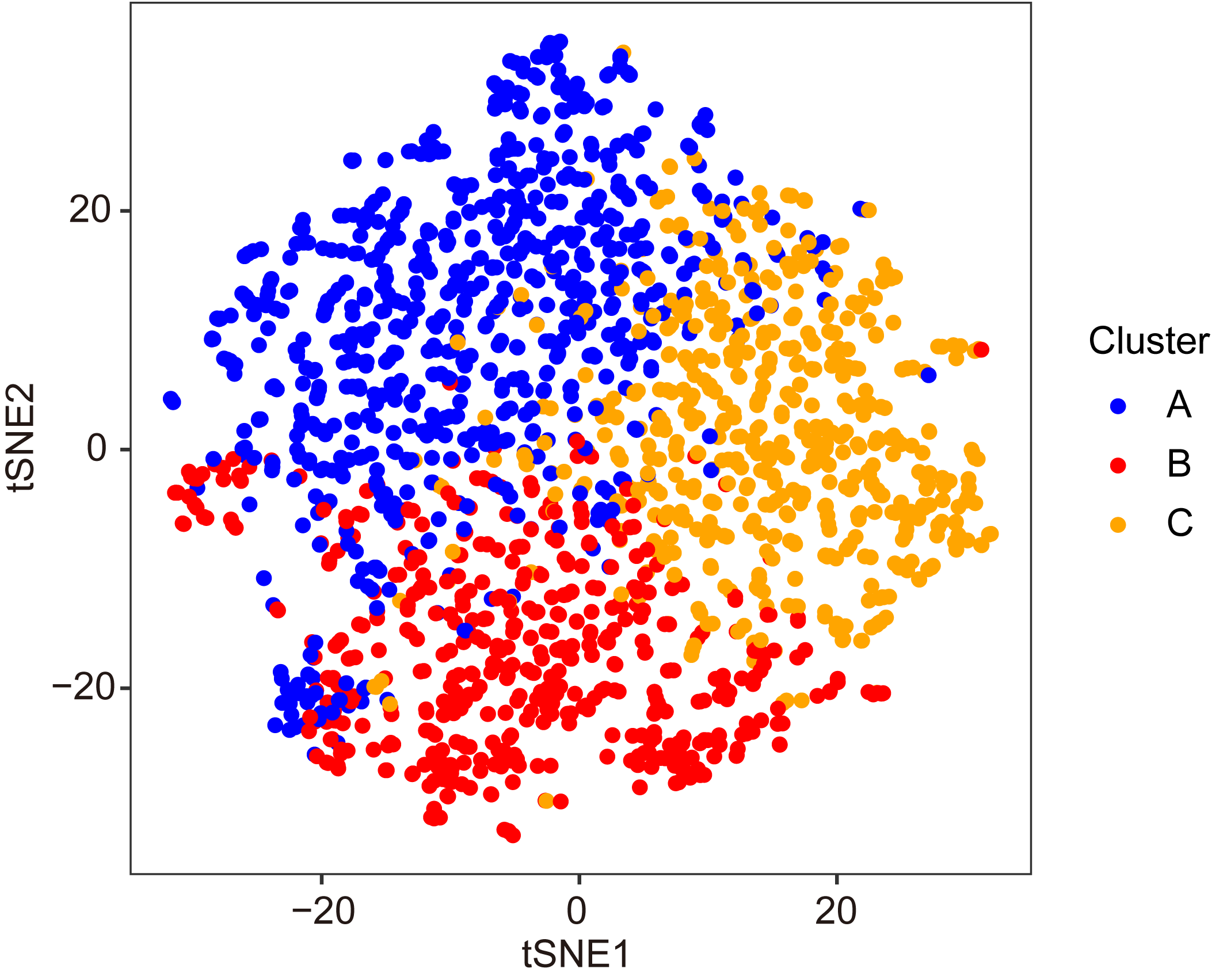

Supplement: Supplementary file 7 [file Image_6.tif]

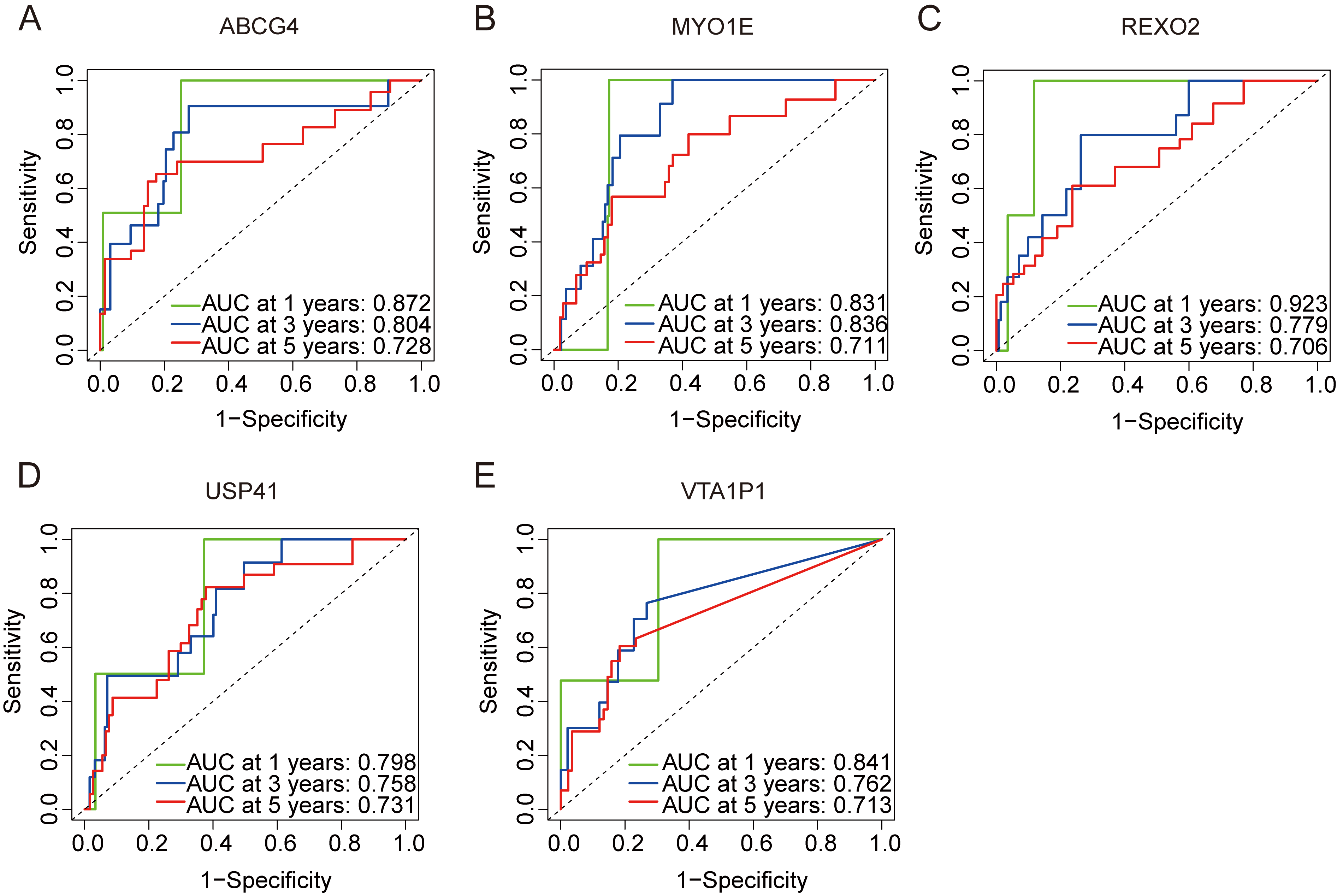

Supplement: Supplementary file 8 [file Image_7.tif]
